# Selection and functional identification of *Dof* genes expressed in response to nitrogen in *Populus simonii* × *Populus nigra*

**DOI:** 10.1515/biol-2022-0084

**Published:** 2022-07-13

**Authors:** Shenmeng Wang, Ruoning Wang, Chengjun Yang

**Affiliations:** Northeast Asia Biodiversity Research Center, Northeast Forestry University, No. 26 Hexing Road, Xiangfang District, Harbin City, Heilongjiang Province, PR China; School of Forestry, Northeast Forestry University, No. 26, Hexing Road, Harbin City, PR China

**Keywords:** Dof transcription factor, *Populus simonii* × *Populus nigra*, transgenic *Arabidopsis thaliana*, carbon and nitrogen metabolism

## Abstract

In plants, Dof transcription factors are involved in regulating the expression of a series of genes related to N uptake and utilization. Therefore, the present study investigated how DNA-binding with one finger (Dof) genes are expressed in response to nitrogen (N) form and concentration to clarify the role of Dof genes and their functions in promoting N assimilation and utilization in poplar. The basic characteristics and expression patterns of Dof genes in poplar were analyzed by the use of bioinformatics methods. Dof genes expressed in response to N were screened, after which the related genes were cloned and transformed into *Arabidopsis thaliana*; the physiological indexes and the expression of related genes were subsequently determined. The function of Dof genes was then verified in *Arabidopsis thaliana* plants grown in the presence of different N forms and concentrations. Forty-four Dof genes were identified, most of which were expressed in the roots and young leaves, and some of the Dof genes were expressed under ammonia- and nitrate-N treatments. Three genes related to N induction were cloned, their proteins were found to localize in the nucleus, and *PnDof30* was successfully transformed into *Arabidopsis thaliana* for functional verification. On comparing *Arabidopsis thaliana* with WT *Arabidopsis thaliana* plants, *Arabidopsis thaliana* plants overexpressing the Dof gene grew better under low N levels; the contents of soluble proteins and chlorophyll significantly increased, while the soluble sugar content significantly decreased. The expressions of several AMT, NRT, and GS genes were upregulated, while the expressions of several others were downregulated, and the expression of PEPC and PK genes significantly increased. In addition, the activity of PEPC, PK, GS, and NR enzymes significantly increased. The results showed that overexpression of *PnDof30* significantly increased the level of carbon and N metabolism and improved the growth of transgenic *Arabidopsis thaliana* plants under low-N conditions. The study revealed the biological significance of poplar Dof transcription factors in N response and regulation of related downstream gene expression and provided some meaningful clues to explain the huge difference between poplar and *Arabidopsis thaliana* transformed by exogenous Dof gene, which could promote the comprehensive understanding of the molecular mechanism of efficient N uptake and utilization in trees.

## 1 Introduction

Nitrogen (N) is one of the several nutrient elements required for the process of plant growth and development [1] and is also one of the most abundant elements in plants. N is present within approximately 70% of the nutrients plants obtain from the environment [2]. N is assimilated into substances that can be used directly or in enzymatic reactions. N is used in various physiological and metabolic reactions, including synthesizing nucleic acids, proteins, coenzyme factors, and molecules involved in signal transmission and storage. However, in nature, the N concentration in the soil is generally low, and N nutrition is often the main factor limiting the growth of plants, including trees [3,4,5]. Fertilization can effectively alleviate N deficiency in an environment in a short period, but it can also reduce the nutrient absorption function of roots [6]; this reduction is not conducive to late plant growth and excessive reliance on chemical fertilizers brings about great pressure to the environment.

DNA-binding with one finger (Dof) transcription factors are plant-specific transcription factors. The Dof family has many members and is part of the zinc-finger protein superfamily. Dof proteins generally range from 200 to 400 amino acids (AAs) in length and contain both a highly conserved N-terminus and a less conserved C-terminus. The N-terminus contains a highly conserved Dof domain of 52 AAs in which the CX2CX21CX2C motif forms a single zinc-finger structure [7]. To date, except for the pumpkin Dof protein AOBP, which recognizes AGTA sequences [8], other Dof proteins recognize AAAG sequences or their reverse complementary sequence CTTT [9,10,11]. The diversity of the C-terminal region of Dofs may be related to the role of different regulatory signals. Combining with different regulatory proteins or other signaling factors to regulate the transcription of target genes is the basis of the functional diversity of Dof transcription factors.

Dof transcription factor-encoding genes generally are members of larger gene families. To date, 37 Dof family members have been identified in *Arabidopsis thaliana* [10], 46 have been identified in maize [12], 30 in rice [13], 26 in barley [11], 28 in soybean [14], 46 in carrot [15], 38 in pea [16], 42 in *Tribulus alfalfa* [17], and 29 in eggplant [18]. Dof transcription factors are largely involved in the regulation of photosynthesis, the synthesis of seed storage proteins, seed development and germination, dormancy, flowering time, cell wall synthesis, the development of vascular bundles, fruit maturity, the accumulation of starch, and other plant-specific biological processes [10,19,20,21,22,23]. In addition, Dof transcription factors also participate in the expression of genes related to the regulation of carbon (C) metabolism [24], the cell cycle [25], abiotic stress tolerance [26,27], and N absorption and utilization [28,29].

In 2000, Yanagisawa performed instantaneous expression in maize protoplasts and used electrophoretic mobility shift assays to identify the target genes regulated by maize Dof1 and found that ZmDof1 could bind the promoter of the phosphoenolpyruvate carboxylase (PEPC) (C4-type PEPC) gene [30]. In 2004, Yanagisawa et al. transformed *ZmDof1* into the C3 plant species *Arabidopsis thaliana*, which improved the N uptake and assimilation efficiency of the transgenic *Arabidopsis thaliana* plants. Under low-N conditions, the transgenic *Arabidopsis thaliana* plants grew better than the wild-type (WT) *Arabidopsis thaliana* plants, and the free AA content significantly increased in the former [31]. Similar results were obtained in 2011 when Kurai transformed *ZmDof1* genes into rice: the N-use efficiency and growth index of the transgenic rice plants significantly improved [32]. In 2008, when Rueda used pine protoplasts to study downstream genes whose expression was regulated by PpDof5 in *Pinus pinaster*, it was found that the transcription factor could activate the expression of the glutamine synthase gene *GS1b* and inhibit the expression of *GS1a* [33]. In 2015, Rueda-López et al. transformed *PpDof5* into *Arabidopsis thaliana* and found that compared with those of the WT plants, the lignin content and C and N metabolism of the transgenic plants significantly increased [34]. *OsDof25*, a functional homolog of *ZmDof1*, was isolated from rice by Santos in 2012, and its expression was determined to be regulated by N. After *OsDof25* was transformed into *Arabidopsis thaliana*, it was found that the C and N metabolism levels improved, and compared with that of the WT plants, the AA content of the transgenic plants significantly increased [35]. Similarly, Wang transformed the *AtDof1* gene into tobacco in 2013 and found that the activities of the PEPC, pyruvate kinase (PK), glutamine synthetase (GS), and nitrate reductase (NR) enzymes increased significantly in the transgenic tobacco compared with the WT [36].

In 2013, however, Lin et al. transformed *ZmDof1* into poplar, but there was no significant change in C and N metabolism or growth between the transgenic poplar and WT poplar [37]. This experiment showed that the N use efficiency of transgenic poplar did not improve under low-N conditions, whether in the culture flask or in the greenhouse, and genes involved in N metabolism and N absorption and utilization, such as PEPC, PK, Asparagine synthetase (AS), GS, NADP-malate dehydrogenase, isocitrate dehydrogenase, and other expression levels did not increase. The promoter regions of the above C/N metabolism-related genes all have Dof binding domain sequence AAAG, indicating that Dof transcription factors recognize these genes. It is possible that the Dof transcription factor in poplar may be involved in regulating the C/N balance pathway, and this regulatory mechanism may be different from that of maize and *Arabidopsis thaliana* function, and screened out the Dof transcription factor that regulates C/N balance in poplar. Therefore, in this study, to identify Dof members in poplar that can improve C and N metabolism and plant growth at low-N levels, we identified the members of the Dof transcription factor family in poplar, identified the genes expressed in response to N through various N treatments, and screened the candidate genes. Sequence analysis was performed, and the subcellular localization of the gene products was subsequently determined. Afterward, the candidate genes were transformed into *Arabidopsis thaliana* growing under low-N levels for functional identification.

In the recent 5 years, various studies on the Dof gene are still emerging one after another. In 2018, Wang screened and identified 24 Dof genes in the Dof genomes of physic nut, and divided them into three categories based on phylogenetic inference. The genome comparison discovered that the expansion of the Dof gene family in physic nut mainly resulted from segmental duplication, and this expansion was mainly subjected to positive selection. Furthermore, many JcDof genes were significantly responsive to the salt and drought treatments [38]. Syed identified Dof transcription factors in pineapple and characterized their expression profiles. Expression analysis using real-time quantitative PCR (qRT-PCR) of pineapple Dof genes family under different abiotic stress (cold, heat, salt, and drought) showed a dynamic response of Dof genes. Thus, we can see that Dof genes expression during abiotic stress reveals their vital role in pineapple growth and development, which could be utilized agronomically [39].

In 2019, Liu et al. investigated the role of PbDof9.2 in flowering regulation in *Pyrus bretschneideri*. It is concluded that the PbDof9.2 suppressed the flowering time regulator FT and could repress flowering time by promoting the activity of PbTFL1a and PbTFL1b promoters. These results suggest that Dof transcription factors have conserved functions in plant development [40]. On the contrary, Tokunaga et al. found that the overexpression of DOF-type transcription factors can enhance lipid synthesis in *Chlorella vulgaris*. Under N-deficient conditions, the transformant CvDOF#3 showed approximately 1.5-fold higher neutral lipid content per cell compared to the original strain and also showed a His-tagged DOF candidate protein expression of 0.6%. Microscopic observations revealed that CvDOF#3 cells were larger. The findings suggested that the overexpression of the endogenous DOF-type transcription factor can be used for improving the lipid content in *Chlorella vulgaris* [41].

In 2020, Waqas conducted a systematic genome-wide analysis of Dof family members in selected cotton species and identified 58, 55, 89, and 110 Dof genes in *G. arboreum*, *G. raimondii*, *G. hirsutum,* and *G. barbadense*, respectively. The combined phylogeny analysis among the GaDof, GrDof, GhDof, GbDof, and AtDof proteins showed orthologous genes among cotton Dofs. This proved the evolution of polyploid cotton from diploid cotton species [42]. In 2021, Neeta analyzed the Dof gene in Brassica napus and concluded that based on the orthology, synteny, and evolutionary analysis, the calculated divergence times indicated that the divergence of the *Brassica* and *Arabidopsis* genus (∼17 Mya), the whole-genome triplication event (9–15 Mya), and the formation of *Brassica napus* (7,500 years ago) drove the expansion of the BnaDof gene family. Synteny analysis also highlighted that most of the Dof genes in *Brassica napus* with known chromosomal locations were not translocated. Tissue-specific expression highlighted the role of BnaDofs in organ development and other developmental processes. Most of the BnaDofs were responsive to temperature fluctuations and were differentially regulated, particularly by cold stress [43].

## 2 Materials and methods

### 2.1 Plant material

Tissue culture-generated seedlings of *Populus simonii* × *Populus nigra* were grown in a growth chamber at 23°C, under 16 h of light and 8 h of darkness, and under a light intensity of 100 μmol m^−2^ s^−1^. Hydroponic cultivation was performed at 25°C, under 16 h of light and 8 h of darkness, and under a light intensity of 120 μmol m^−2^ s^−1^. *Populus simonii* × *Populus nigra* seedlings were cultured in lactate aqueous solution supplemented with 1 mM ammonium-nitrate for 1 week. After 3 days of N being withheld, the N supply was restored for 2 or 48 h, and then the N was withheld again for another 2 or 48 h. The solutions were replaced every 3 days. Samples were collected at each time point and frozen in liquid N for further study.

Seeds of *Arabidopsis thaliana* plants were disinfected in 75% ethanol comprising 0.05% Triton X-100 for 15 min, washed with absolute ethanol, dried, and germinated on 1/2-strength Murashige and Skoog (MS) solid media. An additional 25 mg L^−1^ kanamycin was used for screening the transgenic lines. When the seedlings had developed their first pair of true leaves, they were transplanted into the soil for the eventual harvesting of their seeds. To carry out the hydroponic experiment, a special device was first constructed. The two ends of a 1.5 mL centrifuge tube were removed, and the middle part was filled with 6 g L^−1^ agar and placed on a rectangular plastic plate. An *Arabidopsis thaliana* seed was placed in the center of the agar to germinate, and the device was placed in the liquid nutrient solution, which was an improved version of Hoagland solution (pH = 5.8). Each hydroponic container was placed in 4 L of nutrient solution, which was changed every 3 days. The N concentrations of the nutrient solutions were 0.15, 0.3, and 3 mM (NH_4_NO_3_ and KNO_3_ were at the same molar ratio). Images were collected, and the growth data were statistically significant after 25 and 45 days. Whole plants were then frozen in liquid N for further study.

### 2.2 Gene cloning and vector construction

On the basis of the sequence of *Populus trichocarpa*, we designed the following gene-specific primers for cloning: for *PnDof19*, 5′-AACCAATACTCACTCCTCCAACA-3′ and 5′-AGGGCACATAAAGTAACCAAATC-3′; for *PnDof20*, 5′-AAAGATGATTCAAGAACTCTTAGGA-3′ and 5′-AATTGTTCTTAAGGATATGCACC-3′; and for *PnDof30*, 5′-ACCTGGTCTTTGTCTGTTTACTCTT-3′ and 5′-CTTCCACACCTGTCTTATACCCTTG-3′. Fragment amplification and vector construction involved the use of a KOD Plus Neo high-fidelity DNA polymerase (Toyobo), a pEASY cloning vector (TransGen Biotech), and *Escherichia coli Trans1-T1* sensitive cells (TransGen Biotech).

The plant transient expression vector pBS-GFP was used for subcellular localization. The primers used were as follows: for pBS-*PnDof19*, 5′-agggtaccATGCCGGCAGAATTA-3′ and 5′-tgactagtTTTAAGACCATTCCC-3′; for pBS-*PnDof20*, 5′-agggtaccATGATTCAAGAACTC-3′ and 5′- tgactagtAGGATATGCACCATT-3′; and for pBS-*PnDof30*, 5′-agggtaccATGATTCCTTCGAGA-3′ and 5′-tgactagtAAGAAGTACTGAAGA-3′. The 5′ end of each pair of primers was added to the KpnI and SpeI restriction sites (New England Biolab). *Arabidopsis* plants were genetically transformed using the plant expression vector pROK2, and the primers used for pROK2-*PnDof30* included 5′-actctagaATGATTCCTTCGAGA-3′ and 5′-tgggtaccTCAAAGAAGTACTGA-3′. The 5′ end of the primers was added to the KpnI and SpeI restriction sites (New England Biolab). After restriction enzyme digestion, T4 DNA ligase (TransGen Biotech) was used for fragment ligation. The primers were synthesized and the sequencing was performed by Harbin Boshi Biotechnology.

### 2.3 Genome-wide analysis of the Dof transcription factor family members in *Populus trichocarpa*

The Phytozome (https://phytozome.jgi.doe.gov) [44] and Plant Transcription Factor Database (PlantTFDB) (http://planttfdb.cbi.pku.edu.cn) websites were queried to obtain genome-wide information concerning the Dof transcription factor family in *Populus trichocarpa* [45]. The website of the ProtParam tool (http://web.expasy.org/protparam/) was used to analyze the protein physicochemical properties [46], and the gene structure was analyzed via the Gene Structure Display Server 2.0 website (http://gsds.cbi.pku.edu.cn/) [47]. The WoLF PSORT website was used for subcellular localization predictions (http://www.genscript.com/psort/wolf_psort.html). The chromosome localization of the *Dof* genes was performed based on data from the Phytozome database and from the complete genome sequence of *Populus trichocarpa*, which was obtained in 2006 [48]. ClustalX software was used for multiple comparisons of protein sequences [49], and MEGA 5 software was used to construct phylogenetic trees [50]. Selecton software was used to analyze the evolutionary selection pressure (http://selecton.tau.ac.il) [51], and the mechanistic–empirical combination model was used for the analysis [52]. The AspenDB (http://aspendb.uga.edu/), poplar EFP browser (http://bar.utoronto.ca/efppop/cgi-bin/efpWeb.cgi), and Phytozome websites were used to analyze gene expression patterns, and the MeV 4.7.4 website was used to construct heat maps. The protein sequences of the cloned genes were analyzed with BioEdit multiple comparison software.

### 2.4 PCR, RNA extraction, and qRT-PCR

The RNA extraction reagent used in this experiment was pBIOZOL (Beijing Biomars-Technology). In addition, a PrimeScript^TM^ RT Reagent Kit (TaKaRa) was used for reverse transcription, an SYBR Green Real-time Quantitative Kit (CWBIO) was used to quantify the reagents, and the quantitative PCR instrument used was an ABI 7500 system. The reactions and steps were performed according to the manufacturers’ instructions.

### 2.5 Subcellular localization

PDS-1000 was used for subcellular localization. Microcarriers were bombarded into lower onion epidermal cells. One day after dark culture, the cells were examined via confocal laser microscopy. The nuclei were stained with 4′,6-diamidino-2-phenylindole (DAPI) reagent and imaged under fluorescent light, and combined fields.

### 2.6 Genetic transformation of *Arabidopsis thaliana*

After transforming the pROK2-*PnDof30* vector into *Agrobacterium tumefaciens* GV3101, the gene was transformed into the genotype of *Arabidopsis thaliana* ecotype Col-0 plants by the floral-dip method [53]. The seeds of *Arabidopsis thaliana* homozygous lines were screened on 1/2-strength MS plates supplemented with 25 mg L^−1^ kanamycin for 3 continuous generations.

### 2.7 Measurements of physiological parameters

The chlorophyll, soluble protein, and soluble sugar contents and the activities of PEPC, PK, GS, and NR were determined via standard kits (Suzhou Comin Biotechnology) in accordance with the product instructions.

## 3 Results

### 3.1 Identification and bioinformatic analysis of the Dof transcription factor family members in *Populus trichocarpa*

Dof members usually have a conserved Dof domain. To identify all the Dof members in *Populus trichocarpa*, we searched the *Populus trichocarpa* V3.0 database for the conserved protein sequence from the Phytozome website and ultimately obtained 45 candidate sequences. After comparing the candidate sequences with the *Populus trichocarpa* Dof members in the PlantTFDB, the redundant sequences were removed, and 44 genes that might encode Dof transcription factors were ultimately identified. According to their chromosomal location information, these members were named *PtDof01–44* (Table 1). Among these members, 20 had no introns, 21 had 1 intron, and 3 had 2 introns. The proteins encoded by these genes were 159–506 AAs in length, had a molecular weight ranging from 17.73 to 55.26 kDa, and had an isoelectric point ranging from 4.46 to 10.86. Subcellular localization prediction showed that all the members were localized in the nucleus, except PtDof19 which was localized in the mitochondria.

**Table 1:** Members of the *Dof* gene family in *Populus trichocarpa*

Dof gene	Locus name	Chromosome location	Amino acids	Intron number	Subcellular location	Mass (kDa)	Pi
*PtDof01*	Potri.001G086400	Chr01:6823402… 6825528	285	1	Nucl	31.39	8.27
*PtDof02*	Potri.001G238400	Chr01:24960012… 24961681	332	1	Nucl	35.59	9.85
*PtDof03*	Potri.002G070700	Chr02:4885437…4886585	301	0	Nucl	34.18	4.46
*PtDof04*	Potri.002G129600	Chr02:9717125…9718494	306	1	Nucl	32.22	5.64
*PtDof05*	Potri.002G174300	Chr02:13296746…13298822	263	0	Nucl	28.90	8.93
*PtDof06*	Potri.003G034200	Chr03:4308607…4309970	235	0	Nucl	25.14	8.94
*PtDof07*	Potri.003G144500	Chr03:16086386…16088714	279	1	Nucl	30.68	8.46
*PtDof08*	Potri.004G038800	Chr04:2946651…2948757	304	0	Nucl	33.91	8.55
*PtDof09*	Potri.004G046100	Chr04:3494264…3496215	325	0	Nucl	35.55	9.20
*PtDof10*	Potri.004G046600	Chr04:3543422…3545548	391	1	Nucl	43.13	8.76
*PtDof11*	Potri.004G056900	Chr04:4529874…4530691	159	0	Nucl	17.73	9.28
*PtDof12*	Potri.004G121800	Chr04:11516992…11520601	503	1	Nucl	55.06	5.26
*PtDof13*	Potri.005G131600	Chr05:10630910…10632126	253	0	Nucl	25.99	8.58
*PtDof14*	Potri.005G134200	Chr05:10904734…10906329	331	1	Nucl	35.46	9.77
*PtDof15*	Potri.005G149100	Chr05:13304087…13305115	342	0	Nucl	37.00	8.62
*PtDof16*	Potri.005G188900	Chr05:20644099…20645183	301	0	Nucl	34.04	4.56
*PtDof17*	Potri.006G084200	Chr06:6374602…6376438	326	1	Nucl	34.63	9.02
*PtDof18*	Potri.006G202500	Chr06:21732370…21733236	288	0	Nucl	31.91	6.77
*PtDof19*	Potri.007G036400	Chr07:2818037…2819446	248	0	Mito	25.50	8.37
*PtDof20*	Potri.007G038100	Chr07:2988100…2989665	323	0	Nucl	34.16	8.83
*PtDof21*	Potri.007G058200	Chr07:6232778…6234228	344	0	Nucl	37.08	8.09
*PtDof22*	Potri.008G055100	Chr08:3254228…3256078	345	2	Nucl	36.90	9.09
*PtDof23*	Potri.008G087800	Chr08:5489825…5493164	500	1	Nucl	54.06	6.95
*PtDof24*	Potri.009G029500	Chr09:4021928…4024120	326	1	Nucl	34.65	9.43
*PtDof25*	Potri.010G167600	Chr10:17052686…17055696	496	1	Nucl	54.19	7.28
*PtDof26*	Potri.010G205400	Chr10:19627248…19629172	356	2	Nucl	37.53	9.51
*PtDof27*	Potri.011G047500	Chr11:4061272…4063211	305	0	Nucl	33.89	8.26
*PtDof28*	Potri.011G054300	Chr11:4716002…4717173	325	0	Nucl	35.71	9.35
*PtDof29*	Potri.011G054400	Chr11:4740021…4740708	170	0	Nucl	19.61	10.86
*PtDof30*	Potri.011G055600	Chr11:4863648…4865957	357	1	Nucl	39.20	8.88
*PtDof31*	Potri.011G065900	Chr11:6038802…6040217	165	1	Nucl	18.29	8.82
*PtDof32*	Potri.012G018700	Chr12:1730440…1732794	297	1	Nucl	32.79	7.89
*PtDof33*	Potri.012G063800	Chr12:7683888…7685455	329	0	Nucl	36.23	6.86
*PtDof34*	Potri.012G081300	Chr12:10796766…10799160	312	1	Nucl	34.20	6.90
*PtDof35*	Potri.013G066700	Chr13:5186342…5190516	494	1	Nucl	53.79	6.98
*PtDof36*	Potri.014G036600	Chr14:2984228…2985710	261	0	Nucl	27.53	6.38
*PtDof37*	Potri.014G100900	Chr14:7895311…7897106	229	1	Nucl	25.08	9.21
*PtDof38*	Potri.015G009300	Chr15:611499…613350	255	1	Nucl	28.12	8.69
*PtDof39*	Potri.015G048300	Chr15:5093143…5095147	321	0	Nucl	35.23	7.61
*PtDof40*	Potri.015G077100	Chr15:10212436…10214743	314	1	Nucl	34.79	6.65
*PtDof41*	Potri.016G069300	Chr16:5006439…5007305	225	2	Nucl	25.21	6.72
*PtDof42*	Potri.017G084600	Chr17:10185927…10189438	506	1	Nucl	55.26	5.36
*PtDof43*	Potri.019G040700	Chr19:4633699…4637172	493	1	Nucl	53.50	5.37
*PtDof44*	Potri.T146000	scaffold_455:16693…17845	274	0	Nucl	30.87	4.80

**Figure 1:**
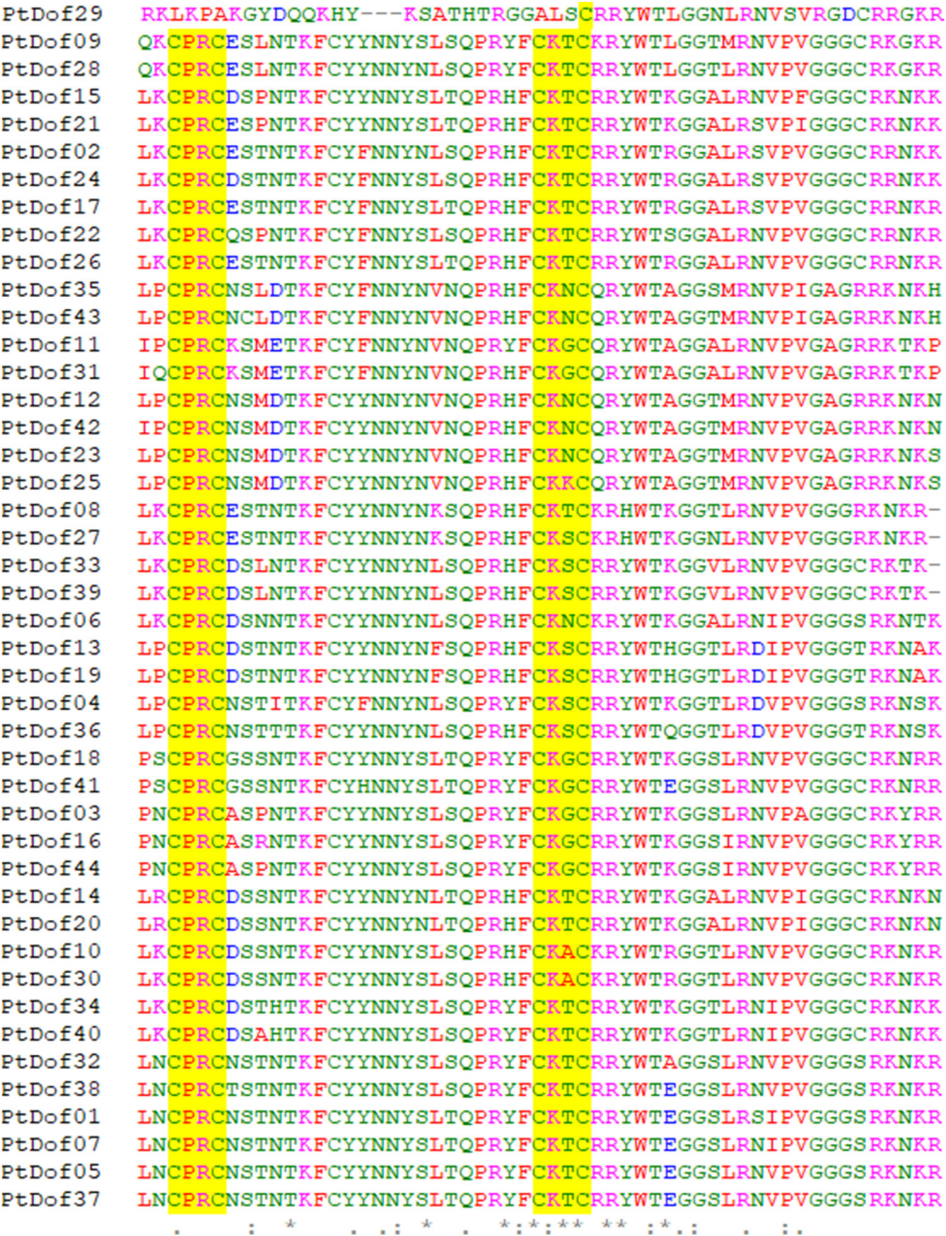
Multiple alignment of 44 conserved PtDof protein sequences. Two sets of four cysteines highlighted in yellow form a zinc-finger structure, and the underlined area is the Dof domain.

Multiple alignments of the Dof domain sequences of the Dof transcription factor members showed that 43 Dof domains were conserved; these domains comprised 54–55 AAs and especially included cysteines at sites 3, 6, 28, and 31. These four cysteines are essential for the formation of zinc-finger structures (Figure 1). Notably, the Dof domain of the PtDof29 protein is incomplete and does not have a typical C2–C2 zinc-finger structure.

**Figure 2:**
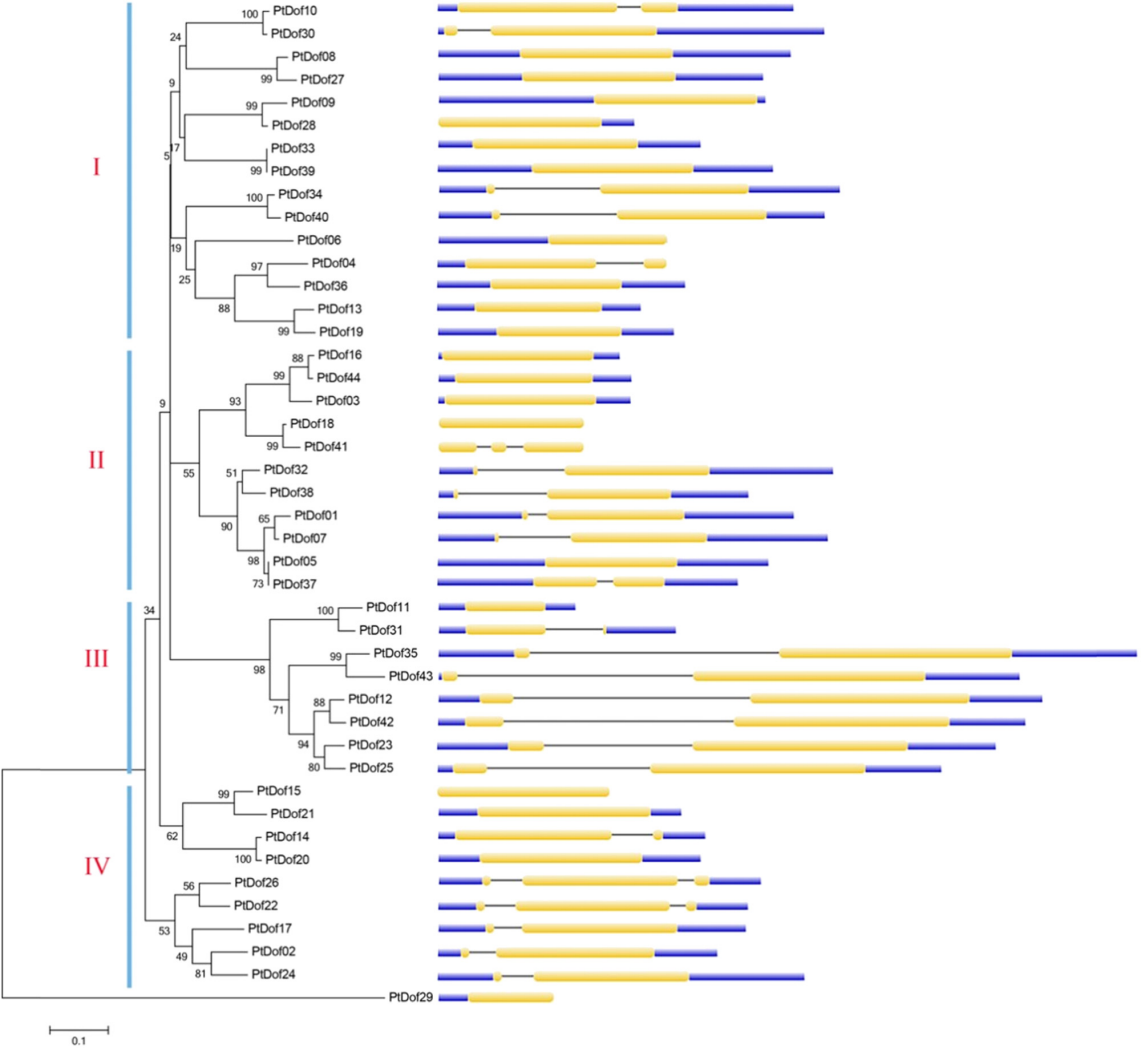
Phylogenetic relationships and gene structure of Dof family members in *Populus trichocarpa*. The NJ phylogenetic tree on the left was constructed via MEGA 5; the tree comprises the aligned protein sequences of 44 PtDof members, and the 4 subfamilies are named I, II, III, and IV. The gene exon/intron structure is shown on the right. The blue lines represent untranslated regions, the yellow lines represent coding areas, and the thin lines represent introns.

To study the evolutionary relationships among members of the Dof family, we constructed a neighbor-joining (NJ) phylogenetic tree of the Dof protein sequences via MEGA 5 software (Figure 2). The results showed that the Dof members of poplar could be divided into 4 subfamilies (I, II, III, and IV) that contained 15, 11, 8, and 9 Dof members, respectively. Although the two branches of the fourth subfamily were not on the same trunk, the evolutionary relationships were very similar between each other, and the gene structures were similar; thus, they were combined into one subfamily. PtDof29 is relatively independent and does not belong to any subfamily. The gene exon and intron structure maps effectively support the classification results of the subfamily.

**Figure 3:**
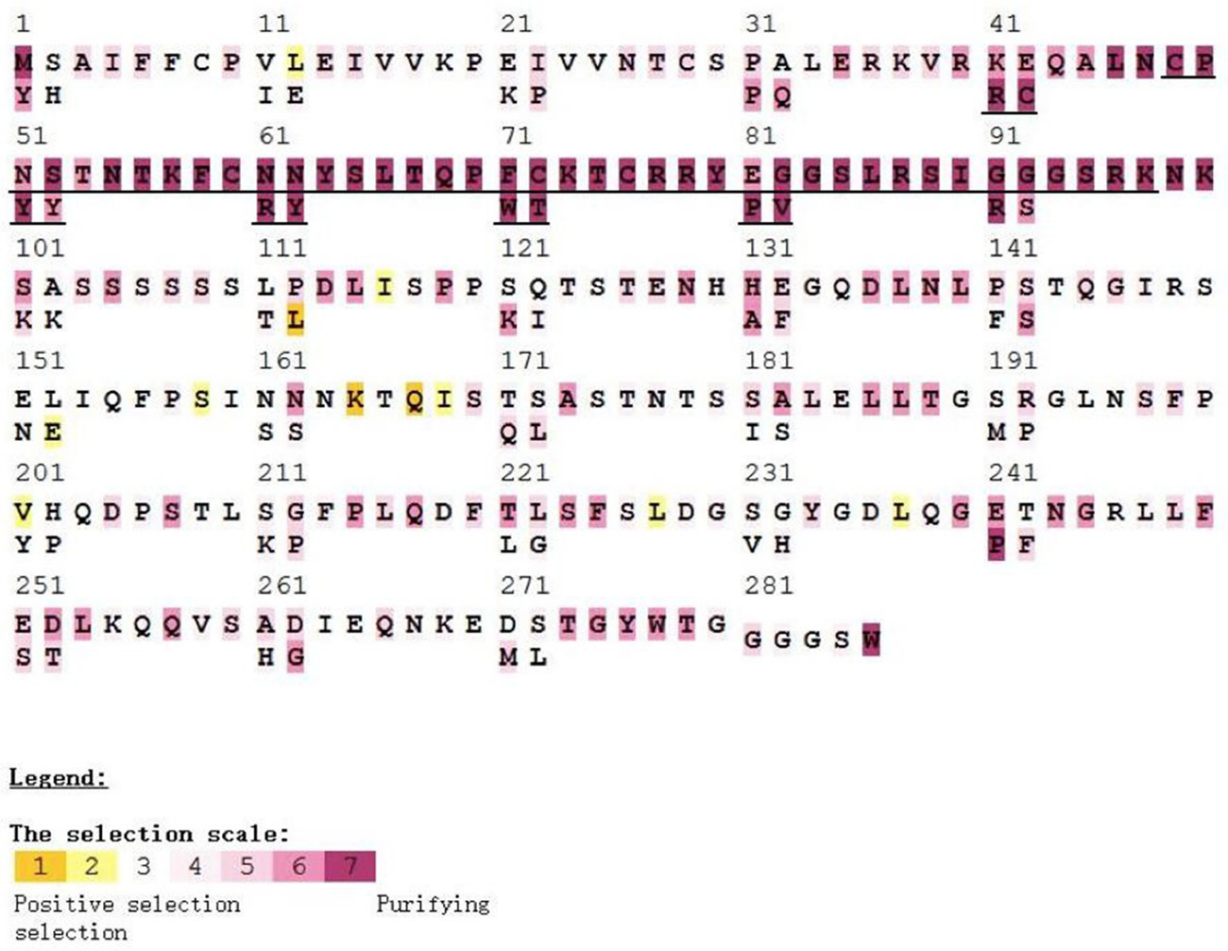
Evolution pressure analysis of *PtDof*s in *Populus trichocarpa*. The black underlined area represents the Dof domain.

The coding DNA sequence (CDS) of the *Populus trichocarpa* Dof gene was input into the Selecton server, and a selection pressure map of each site was obtained (Figure 3). In the maps, yellow represents a positive selection site with less distribution; there was no positive selection site distributed within the Dof domain. White to purple represents negative selection sites; almost all AAs in the Dof domain are associated with a negative selection site, and most of them are dark purple on the map, representing substantial purifying selection. These results indicate that the Dof domain is under a substantial amount of purifying selection, maintaining a high degree of evolutionary rigor; most of the non-Dof domains are under neutral selection. Compared with the other sites, these sites are less conserved and have a higher probability of mutation. This conclusion also explains why the sequences of the Dof family members are quite different.

**Figure 4:**
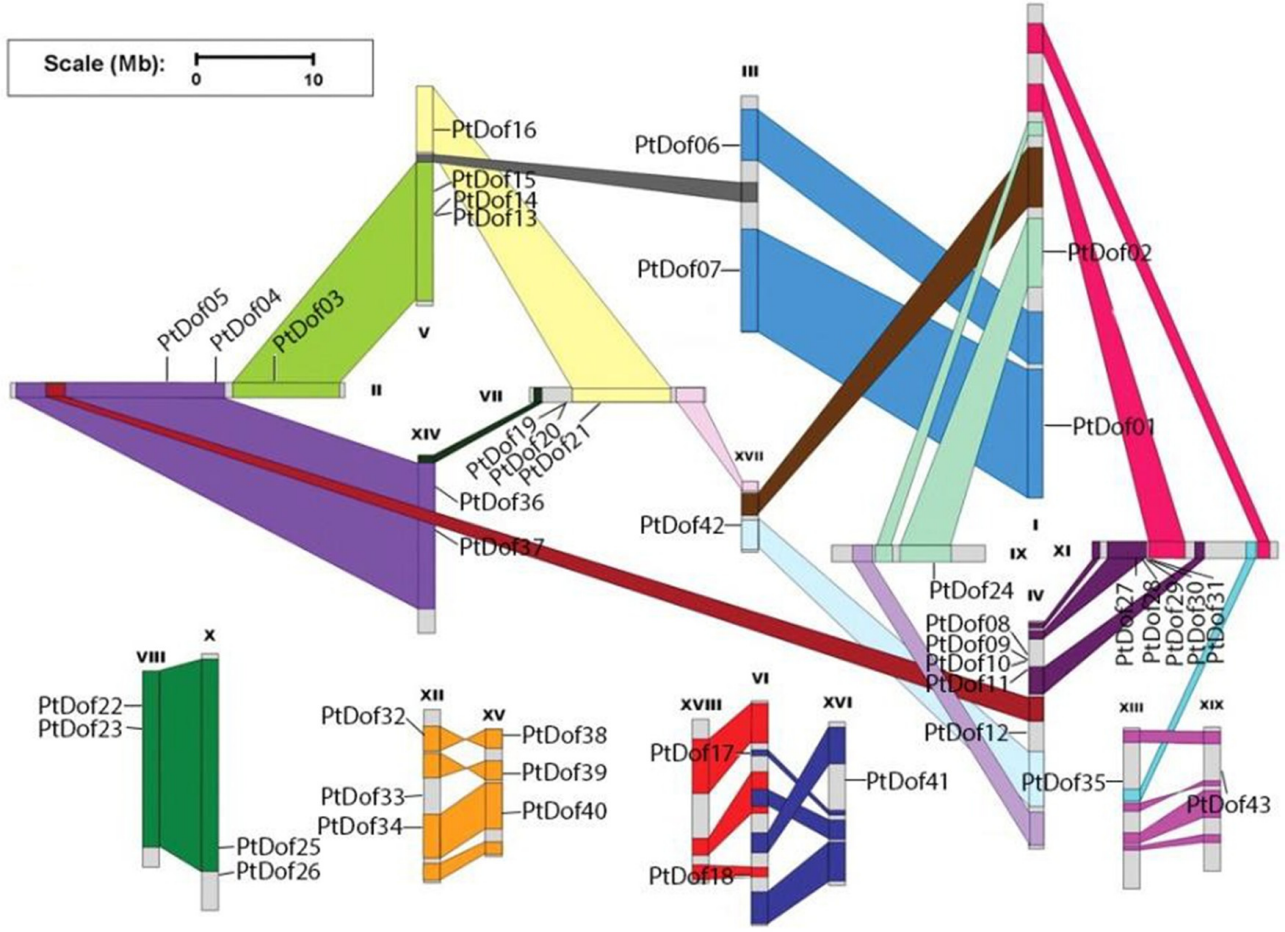
Chromosome mapping of Dof family members in *Populus trichocarpa*. The same colored regions represent chromosomal homologous recombination regions.

Based on the chromosomal location information of the 44 *Dof* genes whose sequence is on the Phytozome website and the homologous recombination map published in 2006, we mapped the chromosomal location map of the *Populus trichocarpa Dof* genes (Figure 4); notably, PtDof29 was unable to be mapped because it belonged to the scaffold structure. The results showed that the Dof genes were distributed throughout the chromosomes, indicating that Dof genes might be ancient. In general, genes within the homologous recombination region of a chromosome may originate from the same ancestor gene. After combining these results with the results of the phylogenetic tree, we identified nine pairs of genes that may have been generated by homologous recombination events of chromosomes in recent evolutionary years: *PtDof1*/*PtDof7*, *PtDof2*/*PtDof24*, *PtDof4*/*PtDof36*, *PtDof5*/*PtDof37*, *PtDof11*/*PtDof31*, *PtDof22*/*PtDof26*, *PtDof23*/*PtDof25*, *PtDof32*/*PtDof38*, and *PtDof34*/*PtDof40*. These nine gene pairs were present not only in the homologous recombination region of the same chromosome but also in the same branch of the evolutionary tree. Among the Dof genes in *Populus trichocarpa*, these nine pairs are most homologous. Therefore, we speculate that these nine pair of genes may have originated from homologous recombination events throughout the evolution.

**Figure 5:**
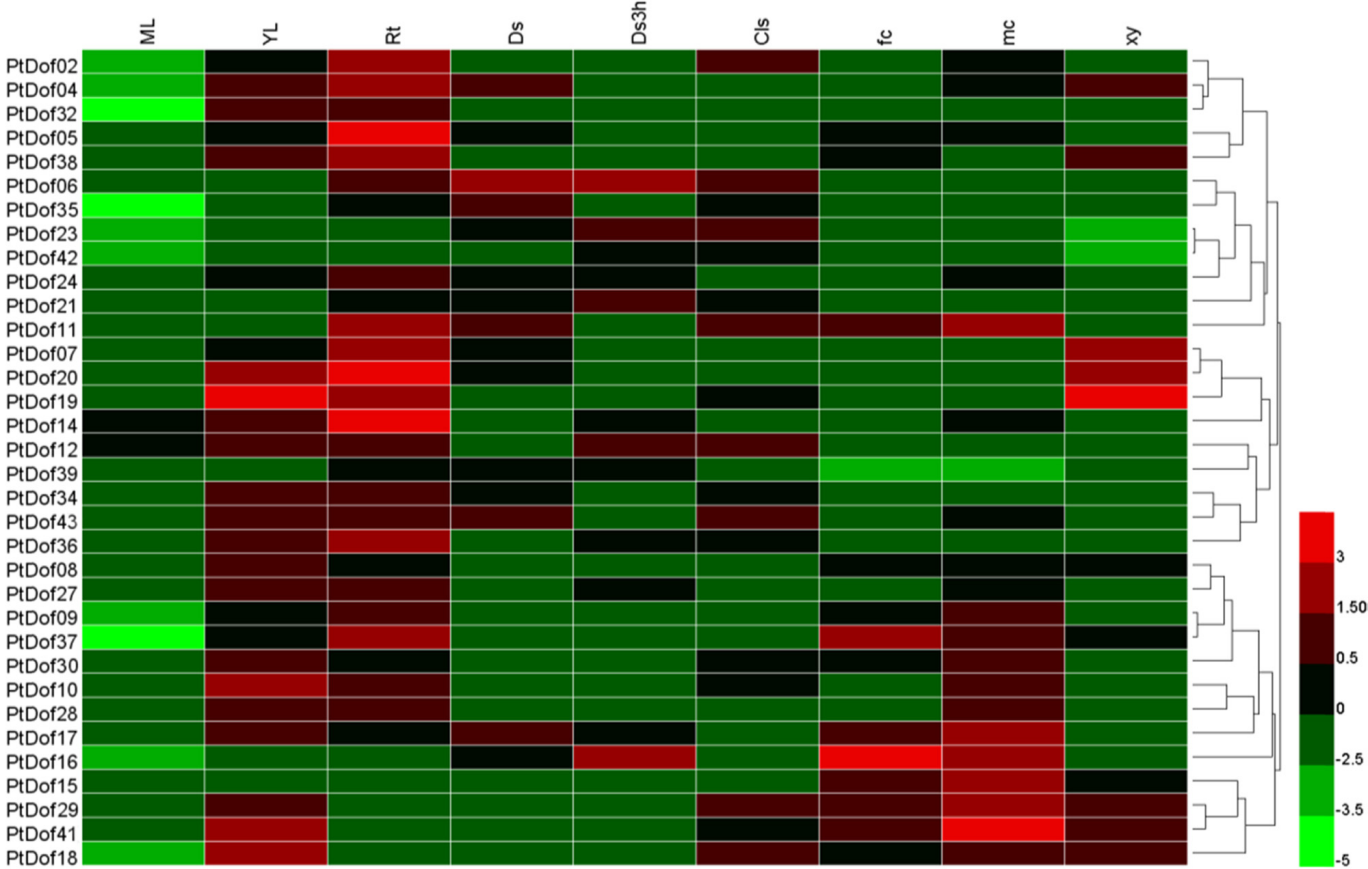
Expression patterns of 34 *Populus trichocarpa* Dof members based on EFP data. ML: Mature leaves; YL: Young leaves; Rt: Roots; Ds: Dark-grown seedlings; Ds3h: Dark-grown seedlings exposed to light for 3 h; Cls: Continuous light-grown seedlings; fc: Female catkins; mc: Male catkins; xy: Xylem.

After searching the AspenDB website for sequences of gene probes for the *Populus trichocarpa* Dof family members, we searched the expression data of each gene in the EFP database. In total, 34 Dof gene expression data points were ultimately identified and used to construct an expression map (Figure 5). The results showed that the expression of the *Dof* genes was mostly downregulated in the mature leaves. However, in the young leaves, the expressions of 9 genes was downregulated, and the expressions of the other 25 genes were upregulated. The expressions of the PtDof10, PtDof18, PtDof20, and PtDof41 genes were relatively high, and the expression of the PtDof19 gene was the highest. In the roots, the expressions of 7 genes was downregulated, and that of 27 genes were upregulated, of which the expressions of PtDof5, PtDof14, and PtDof20 were the highest. In the young leaves of plants growing in the darkness, the expressions of 15 genes was upregulated, and that of 19 genes were downregulated. In the young leaves of plants growing in darkness but then exposed to light for 3 h, the expressions of 12 genes was upregulated, and that of 22 genes were downregulated. In seedlings subjected to continuous light, the expressions of 17 genes were upregulated, and that of 17 genes were downregulated. In the female flowers, the expressions of 13 genes were upregulated, and that of 21 genes were downregulated, and in the male flowers, the expressions of 20 genes were upregulated, and that of 14 genes were downregulated. In the xylem, the expressions of 11 genes was upregulated, and that of 33 genes were downregulated. In conclusion, most of the members of the *Populus trichocarpa* Dof gene family were expressed in young leaves and roots, and the expression patterns in other plant parts were more complex, which indicated that the function of Dof genes might be substantially different.

**Figure 6:**
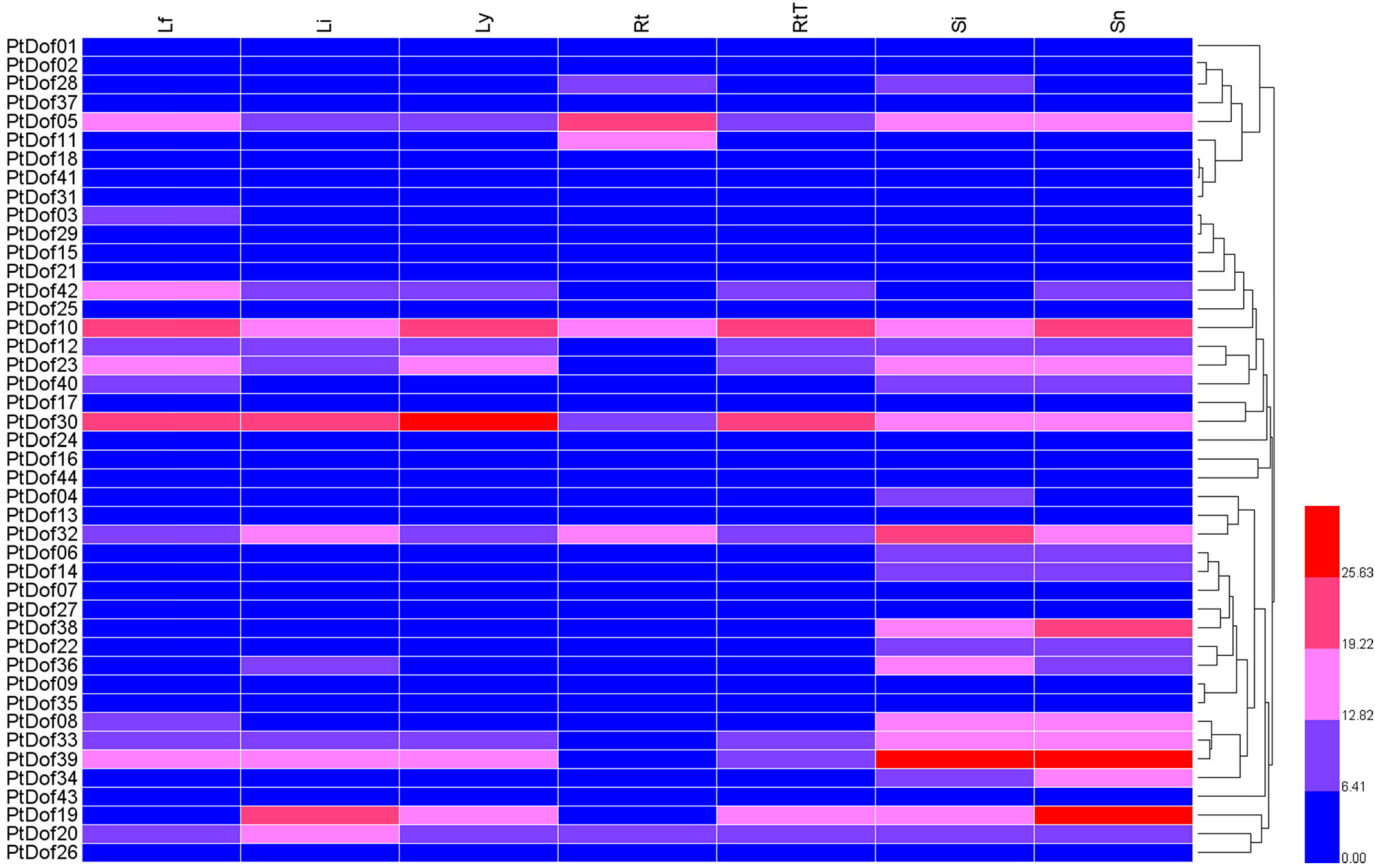
Expression patterns of 44 *Populus trichocarpa* Dof members based on FPKM data obtained from the Phytozome website. Lf: Leaves (early-stage female floral buds); Li: Leaves (immature); Ly: Leaves (young); Rt: Roots; RtT: Root tips; Si: Stems (internodes); Sn: Stems (nodes).

The fragments per kilobase of transcript per million mapped reads (FPKM) data of the *Populus trichocarpa Dof* genes were obtained from the Phytozome website, and the expression data were used to construct a gene expression heat map (Figure 6). The results showed that the expressions of *PtDof10* and *PtDof30* were higher in the leaves (early stage of female floral buds) than in the other organs. In the leaves (immature ones), the most highly expressed genes were *PtDof30* and *PtDof19*. In the young leaves, *PtDof10* and *PtDof30* were highly expressed; in the roots, the *PtDof5* gene was expressed the most. The most highly expressed genes in the root tips were *PtDof10* and *PtDof30*. In the stems (internodes), the most highly expressed genes were *PtDof32* and *PtDof39*; in the stem nodes, the most highly expressed genes were *PtDof19*, *PtDof10*, *PtDof38*, and *PtDof39*. The expression of *PtDof05*, *PtDof10*, *PtDof19*, *PtDof20*, *PtDof23*, *PtDof30,* and *PtDof39* in various tissues was significantly higher than that of the other studied genes.

### 3.2 Screening of Dof genes in response to N changes in *Populus simonii* × *Populus nigra*

**Figure 7:**
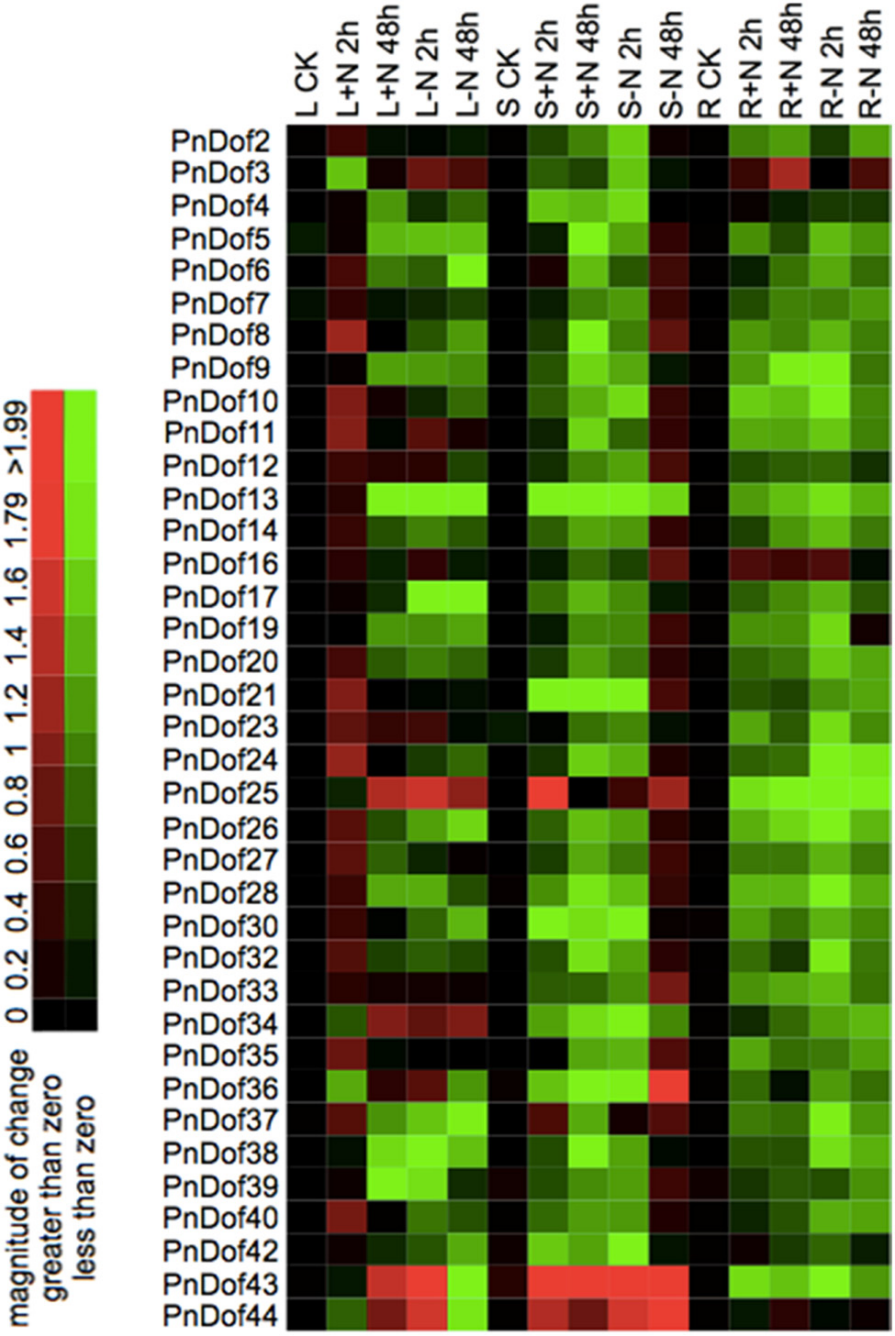
Relative expression levels of the *Populus simonii* × *Populus nigra PnDof* genes under sufficient and deficient N supplies, as revealed by qRT-PCR (L: leaves; S: stems; R: roots).

Two groups of tissue culture-generated seedlings were subjected to N-treatment experiments under LA hydroponic solution. In the first group, 1 mM ammonium-nitrate was used as the sole N source (Figure 7). After 1 week of cultivation, the seedlings were subjected to an N-deficient solution for 3 days. Afterward, they were subjected to an N-sufficient solution for 2 or 48 h, after which the N was withheld again for 2 or 48 h. Samples were taken at each of these time points. *Populus simonii* × *Populus nigra* seedlings were grown under N-deficient conditions *in vitro* for 3 days as controls and were provided different forms of N *in vivo* only to maintain biological activity. After the N was absorbed under *in vivo* conditions, the N treatments were carried out *in vitro* to obtain information on *Dof* genes induced in response to N in *Populus simonii* × *Populus nigra*. qRT-PCR was used to measure the expression of the Dof genes (the primers used are listed in Table 1). The internal reference gene used was cell division control protein 2 (CDC2), and the 2^−ΔΔCt^ method was used to calculate the relative expression. After several rounds of designing primers and performing quantitative experiments, the expression data of 38 genes were ultimately obtained. The results showed that the expression patterns of these genes in the leaves, stems, and roots were very different under different treatments, showing different degrees of tissue specificity. Compared with that in the control group, the expressions of 31 Dof genes in the leaves of the treatment group increased 2 h after the N supply was restored. After the N supply was restored for 48 h, the expression levels were lower than that after the N supply was restored for 2 h, and the expression levels of most of the *Dof* genes were lower after N was withheld than when the N was supplied for 2 and 48 h. It could be concluded that a short N supply induces the expressions of most *Dof* genes in the leaves but that a prolonged N supply inhibits the expressions of some of these genes. In the stems, 32 *Dof* genes presented higher expression levels when N was withheld for 48 h but lower expression levels when N was resupplied for 2 and 48 h. These results suggest that low N contents *in vivo* induced the expressions of *Dof* genes in the absence of an N supply *in vitro*. In the roots, the expressions of 34 *Dof* genes decreased 2 h after the N was resupplied, while the expressions of 17 *Dof* genes increased slightly within the 48 h during which the N was resupplied and withheld again. This may indicate that the expressions of some Dof genes was induced under low N levels in the roots.

**Figure 8:**
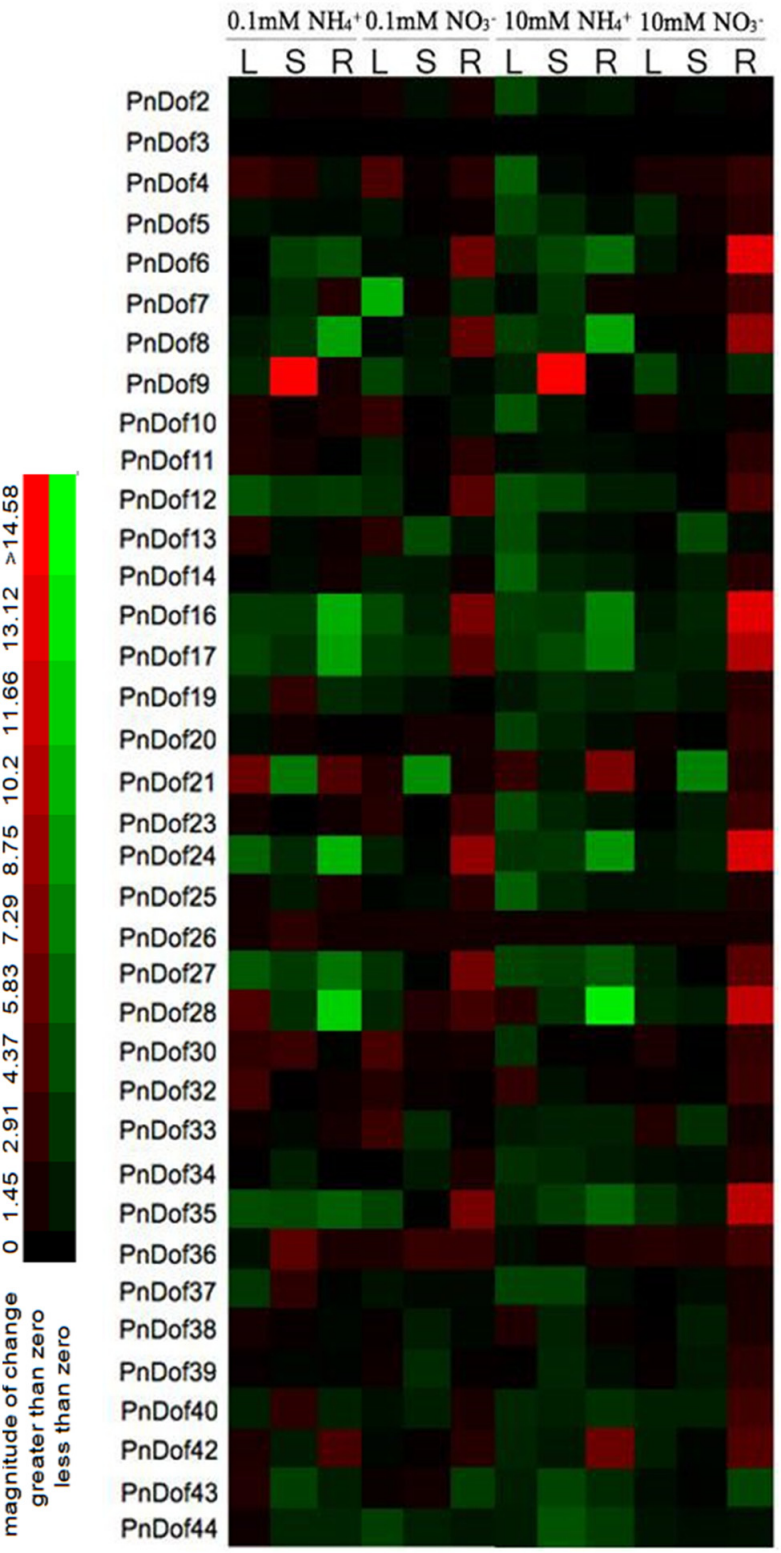
Relative expression levels of *Populus simonii* × *Populus nigra PnDof* genes under two kinds of N supplied at three different concentrations, as revealed by qRT-PCR (L: Leaves; S: Stems; R: Roots).

In the second N treatment experiment, seedlings were treated with ammonium and nitrate N for 2 weeks at concentrations of 0.1, 1, or 10 mM (Figure 8). The results showed that the expressions of ten genes (*Dof4*, *Dof10*, *Dof11*, *Dof13*, *Dof21*, *Dof28*, *Dof30*, *Dof32*, *Dof42,* and *Dof43*) were induced in the leaves under low ammonium concentrations, that of eight genes (*Dof4*, *Dof9*, *Dof19*, *Dof26*, *Dof30*, *Dof36*, *Dof37*, and *Dof40*) were induced in the stems, and that of seven genes (*Dof7*, *Dof9*, *Dof10*, *Dof21*, *Dof25*, *Dof36,* and *Dof42*) were induced in the roots. Under low-nitrate conditions, the expressions of nine genes were induced in leaves, including *Dof4*, *Dof10*, *Dof13*, *Dof21*, *Dof23*, *Dof30*, *Dof32*, *Dof33*, and *Dof36*; the expressions of four genes were induced in stems, including *Dof26*, *Dof28*, *Dof36*, and *Dof43*; and the expressions of 19 genes were induced in the roots. In conclusion, the expressions of the following genes was induced under both low levels of N at the same time: *Dof4*, *Dof10*, *Dof13*, *Dof21*, *Dof30*, and *Dof32* in leaves; *Dof36* in the stems; and *Dof25*, *Dof36*, and *Dof42* in the roots.

### 3.3 Cloning of the *PnDof19*, *PnDof20*, and *PnDof30* genes from *Populus simonii* × *Populus nigra*

**Figure 9:**
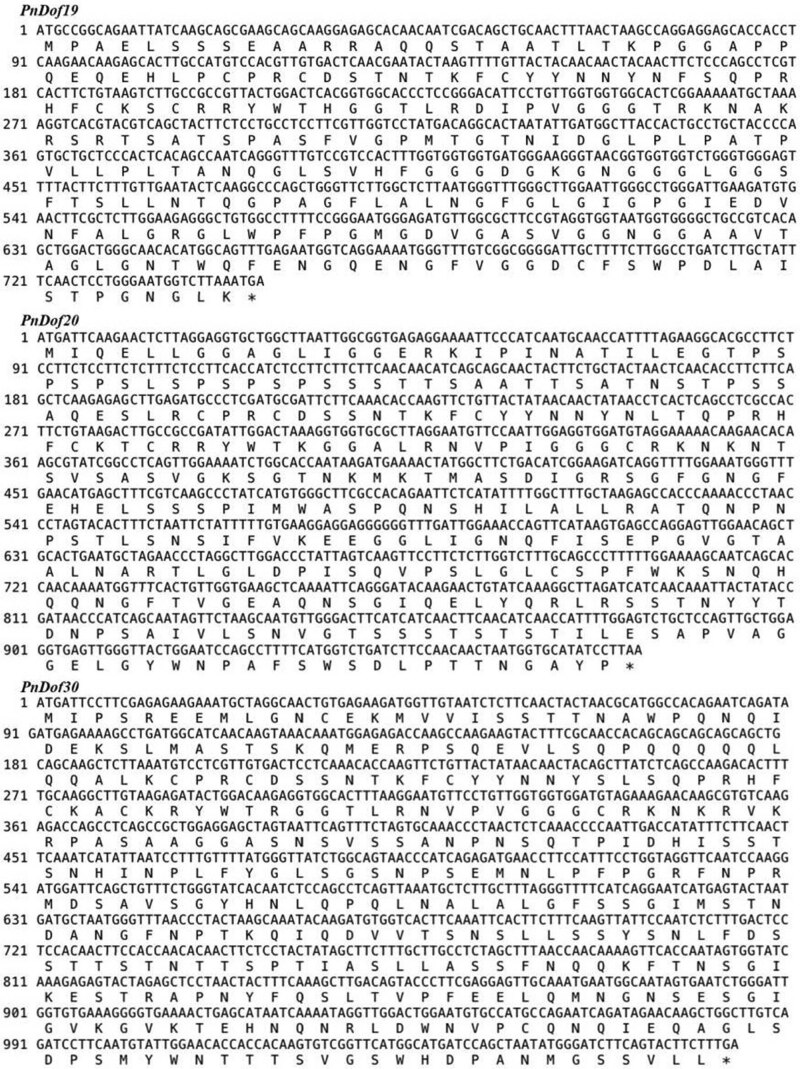
Gene coding sequences of *PnDof19, PnDof20*, and *PnDof30* alongside their translated protein sequences in *Populus simonii × Populus nigra*.

We cloned the CDSs of the *PnDof19* (MK796000), *PnDof20* (MK7960001), and *PnDof30* (MK789595) genes from *Populus simonii* × *Populus nigra* cDNA (Figure 9), which were 747, 972, and 1,074 bp long, respectively, and encoded 248, 323, and 357 AAs, respectively.

**Figure 10:**
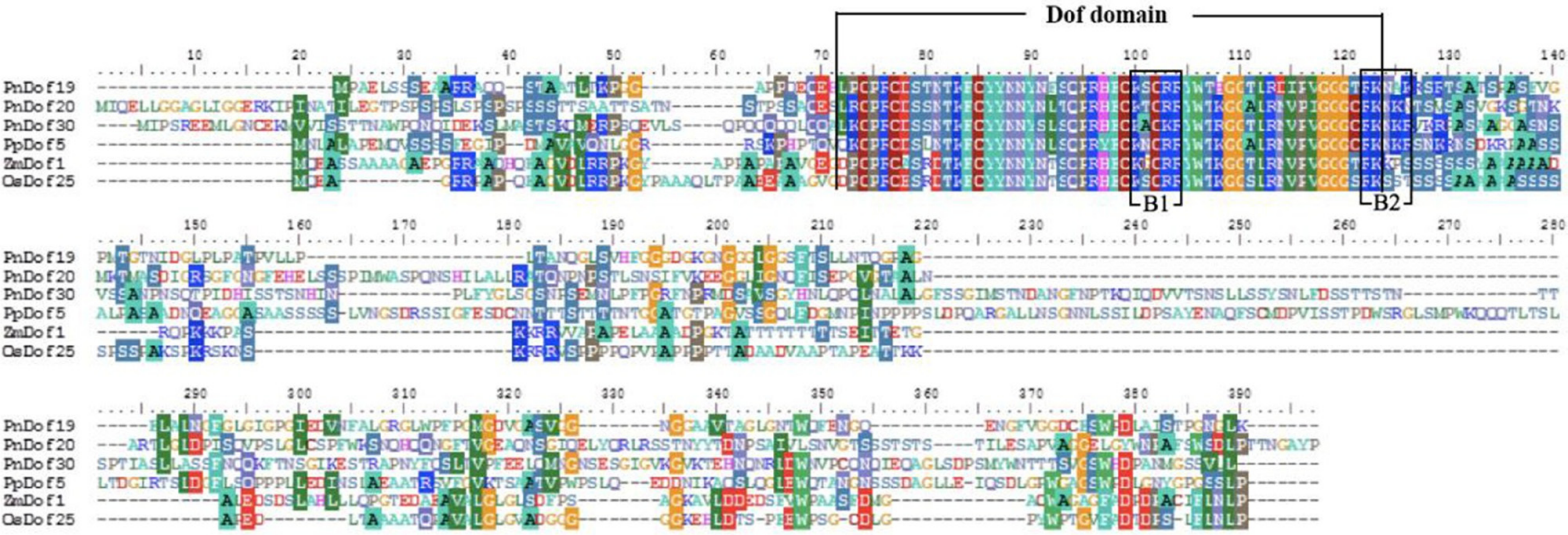
Multiple alignment of protein sequences. The black box area is the conserved Dof domain, and B1 and B2 represent Dof-specific nuclear localization signals.

To explore whether the transcription factors encoded by the *PnDof19*, *PnDof20,* and *PnDof30* genes cloned from *Populus simonii* × *Populus nigra* are involved in regulating C and N metabolism, we compared the sequences of these three proteins with the sequences of three functional proteins that specifically regulate C and N metabolism (Figure 10). The results showed that all six proteins had a complete Dof domain and four highly conserved cysteines, and all of them had a nuclear localization signal specific to Dof transcription factor family members (B1 and B2 regions).

**Figure 11:**
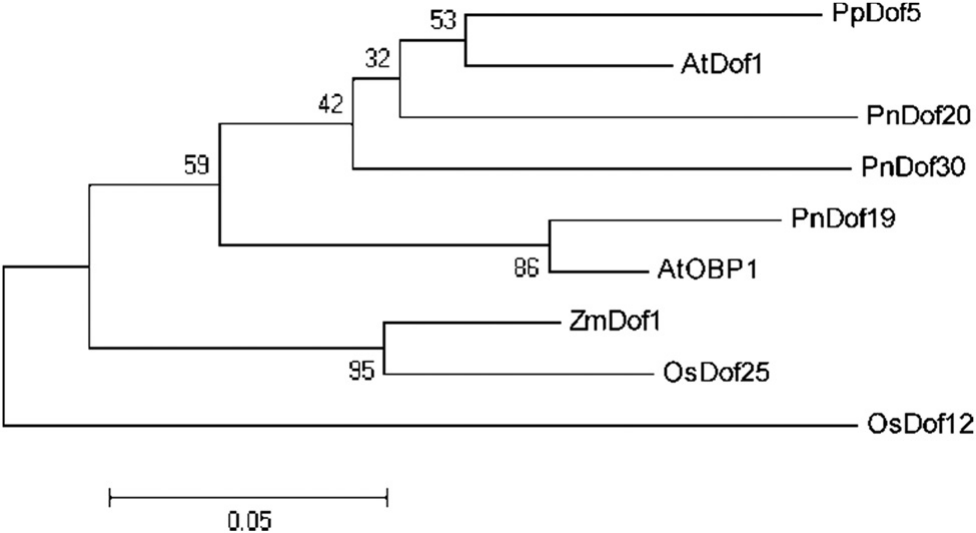
Phylogenetic tree comprising *PnDof19*, *PnDof20*, and *PnDof30* in *Populus simonii × Populus nigra* and several specific functional *Dof* genes in other species.

Moreover, we identified Dof genes with different functions (both *AtDof1*/NM_104048.4 and *AtOBP1*/OAP05220.1 in *Arabidopsis thaliana* and *OsDof12*/AAL84292.1 in rice) and constructed phylogenetic trees comprising the CDSs of both the three protein-coding genes cloned by us and the known Dof protein-coding genes (Figure 11). Because of the poor conservation of Dof protein sequences of non-Dof-domain regions, we used only conserved domain sequences to construct phylogenetic trees to determine their evolutionary relationships more accurately. The results showed that *PnDof20* and *PnDof30* branched together with the N metabolism regulatory genes *AtDof1* and *PpDof5*, suggesting that *PnDof20* and *PnDof30* may also be N metabolism regulatory genes; *PnDof19* and the cell cycle regulatory gene *AtOBP1* were branched together on one branch, and thus, we speculated that *PnDof19* might be related to cell cycle regulation. *OsDof12* is a flowering regulatory gene that is independent of the other genes in the phylogenetic tree.

### 3.4 Subcellular localization of the *PnDof19*, *PnDof20*, and *PnDof30* proteins

**Figure 12:**
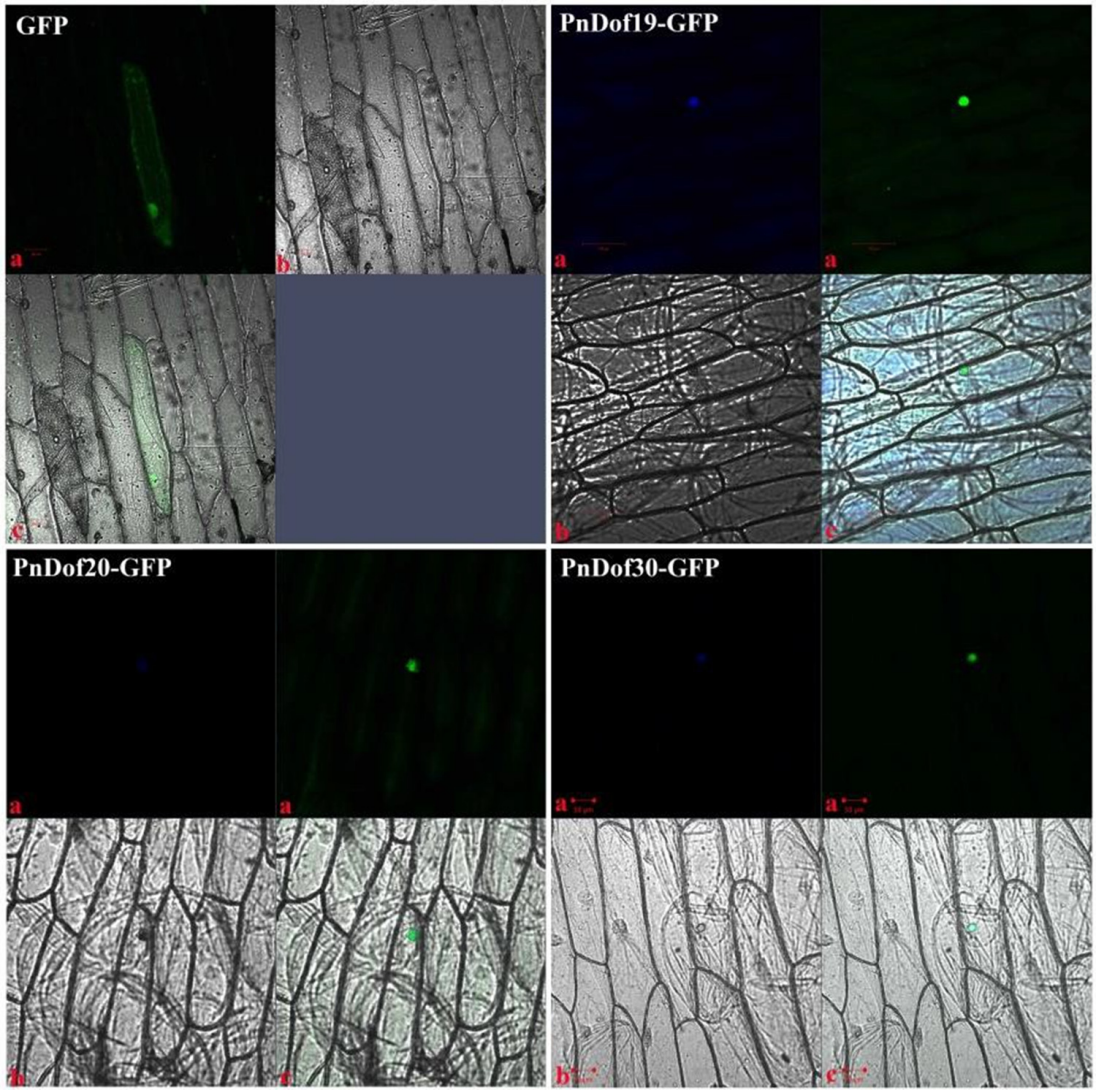
Subcellular localization of the *PnDof19*, *PnDof20*, and *PnDof30* proteins. a: Fluorescence field; b: Bright field; and c: Superimposition of the fluorescence and bright fields.

To determine whether the three *Populus simonii* × *Populus nigra* Dof proteins have characteristics of general transcription factors, i.e., the localization of the protein in the nucleus, we fused the open reading frame of the *PnDof19*, *PnDof20,* and *PnDof30* genes to the *GFP* gene within a PBS-*GFP* vector; onion subepidermal cells were subsequently transformed via gene gun bombardment. Cells displaying green fluorescence were observed via scanning laser confocal microscopy. The nuclei were stained with DAPI reagent and then observed and imaged under a microscope (Figure 12). The results showed that the *PnDof19*, *PnDof20*, and *PnDof30* proteins localized to the nucleus, which is consistent with the localization of Dof proteins in other reported species [54,55].

### 3.5 Functional analysis of *PnDof30*-overexpressing *Arabidopsis thaliana* lines

**Figure 13:**
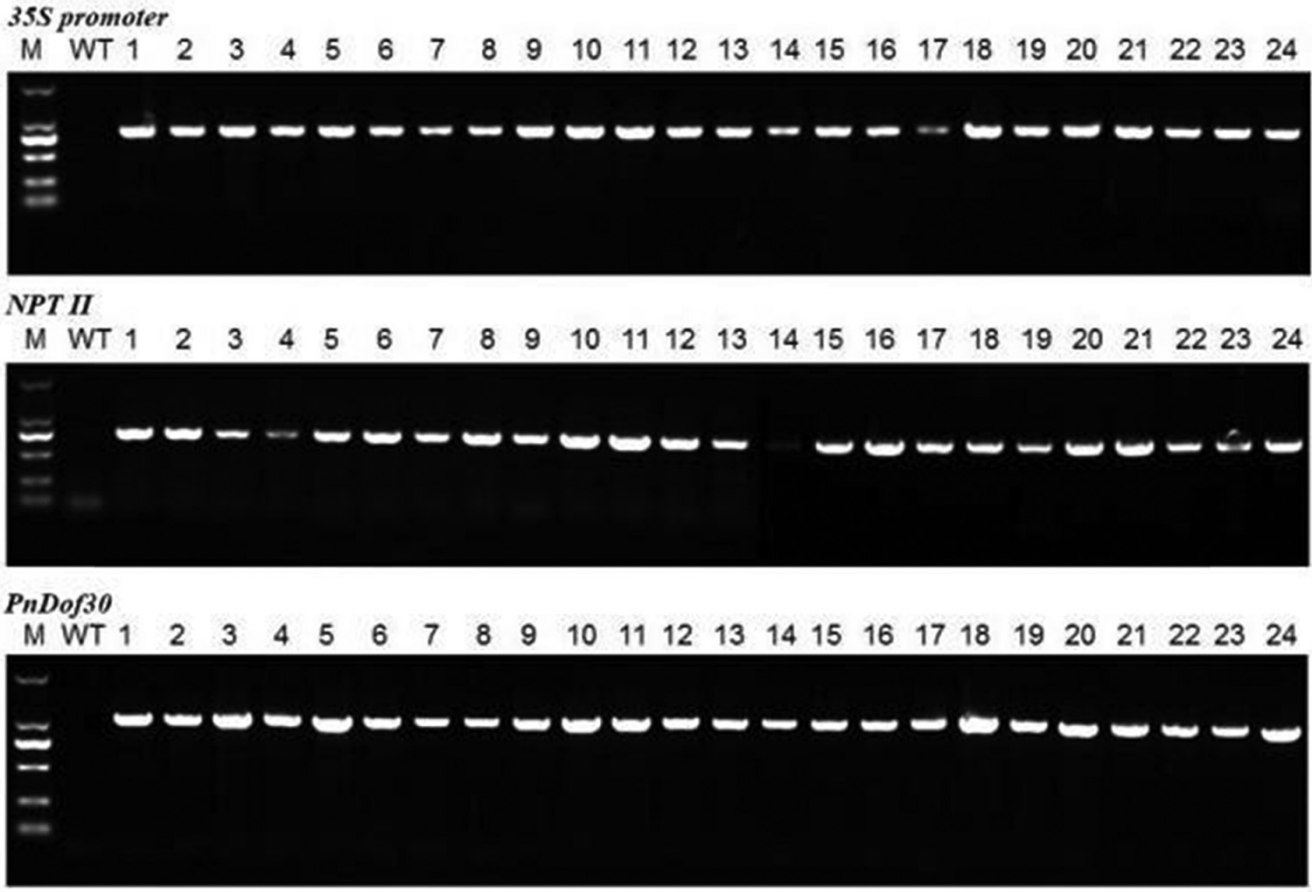
PCR-based identification of genomic DNA in transgenic *Arabidopsis thaliana* lines.

**Figure 14:**
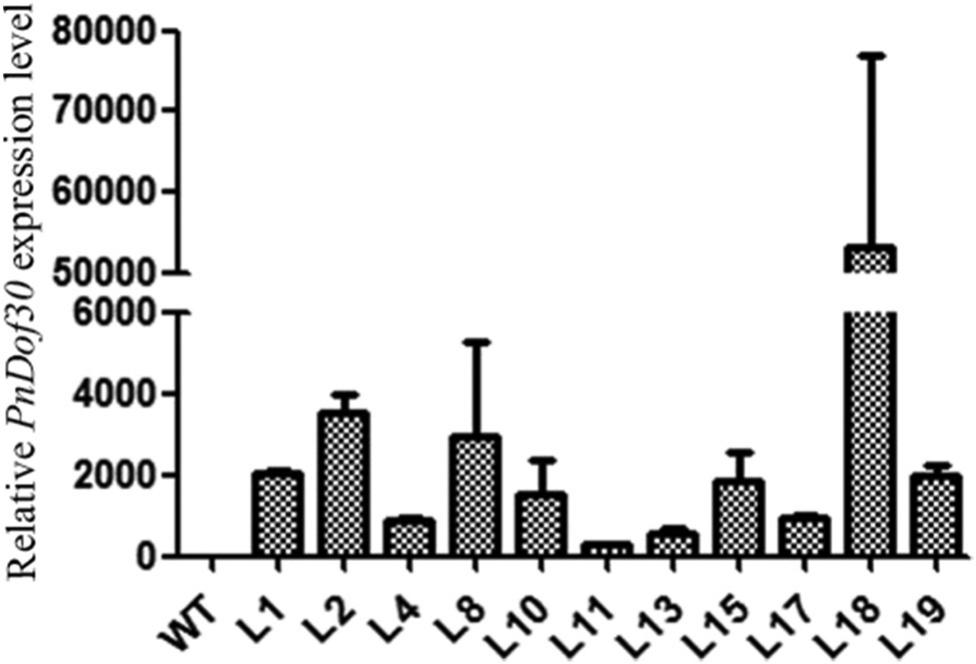
Relative expression of *PnDof30* in transgenic *Arabidopsis thaliana* lines.

The *PnDof30* gene was inserted into the genome of *Arabidopsis thaliana* ecotype Col-0 plants by the floral-dip method, and 24 independent transgenic lines were selected for extraction of their genomic DNA. The transgenic lines were identified via PCR (Figure 13). After identification, 11 transgenic lines were randomly selected to determine the expression level of their *PnDof30* gene (Figure 14). The results showed that the expression level and stability in each line were substantially different. We chose three stable expression lines, L1, L2, and L15, for functional analysis and then screened and identified the homozygotes.

**Figure 15:**
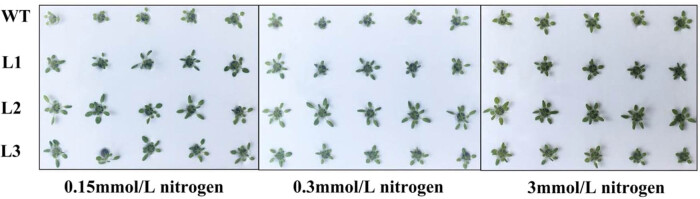
Phenotypic changes in *PnDof30* transgenic *Arabidopsis thaliana* lines under N treatment for 25 days.

The seeds of the WT and homozygous lines were vernalized and planted in several unique hydroponic devices for germination and growth. The hydroponic solutions used were improved versions of Hoagland solution, consisting of 0.15, 0.3, or 3 mM N. The phenotypes were evaluated after 25 days of plant growth (Figure 15). The results showed that the phenotypes of the growth-related parameters of the *PnDof30*-overexpressing *Arabidopsis thaliana* lines, such as leaf size, lotus leaf diameter, and leaf number, were significantly better than those of WT plants under all three different N concentrations, especially under low-N conditions.

**Figure 16:**
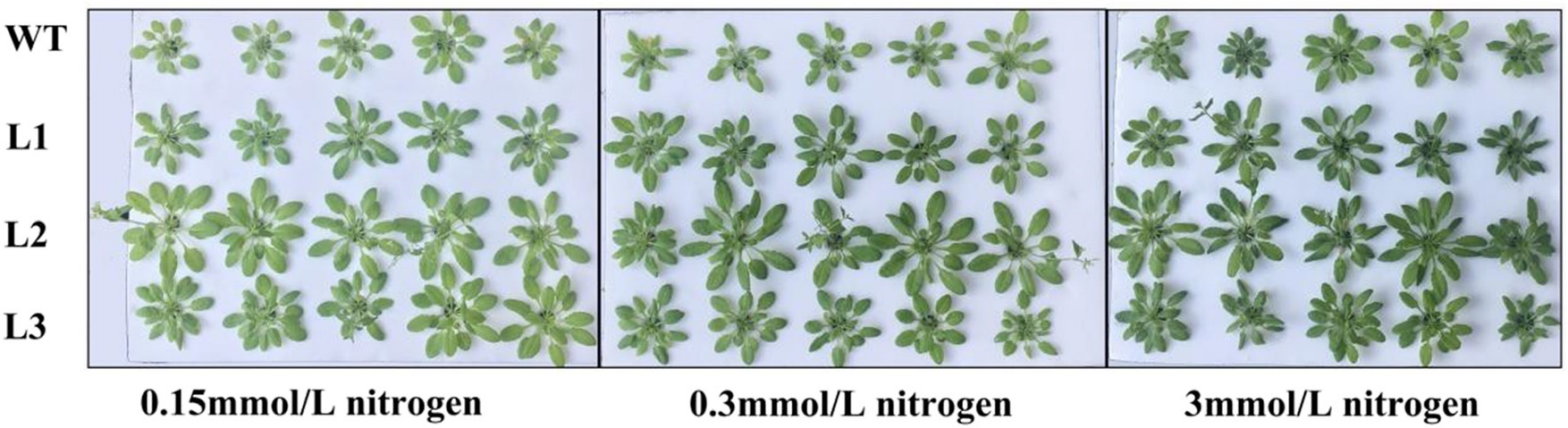
Phenotypic changes in *PnDof30* transgenic *Arabidopsis thaliana* lines under N treatment for 45 days.

Because the plant size was too small for further determination of growth-related parameters, we cultured *Arabidopsis thaliana* plants in liquid media for 45 days and observed their phenotype (Figure 16). The results showed that when the culture period was extended to 45 days, the overexpression plants under low-N conditions still grew better than WT plants, and the leaf color was greener than that of WT plants. Under 3 mM N, the difference in growth between the overexpression lines and WT narrowed; nonetheless, the L2 transgenic lines were significantly better than the WT, and L1 and L3 transgenic lines were slightly better than the WT.

**Figure 17:**
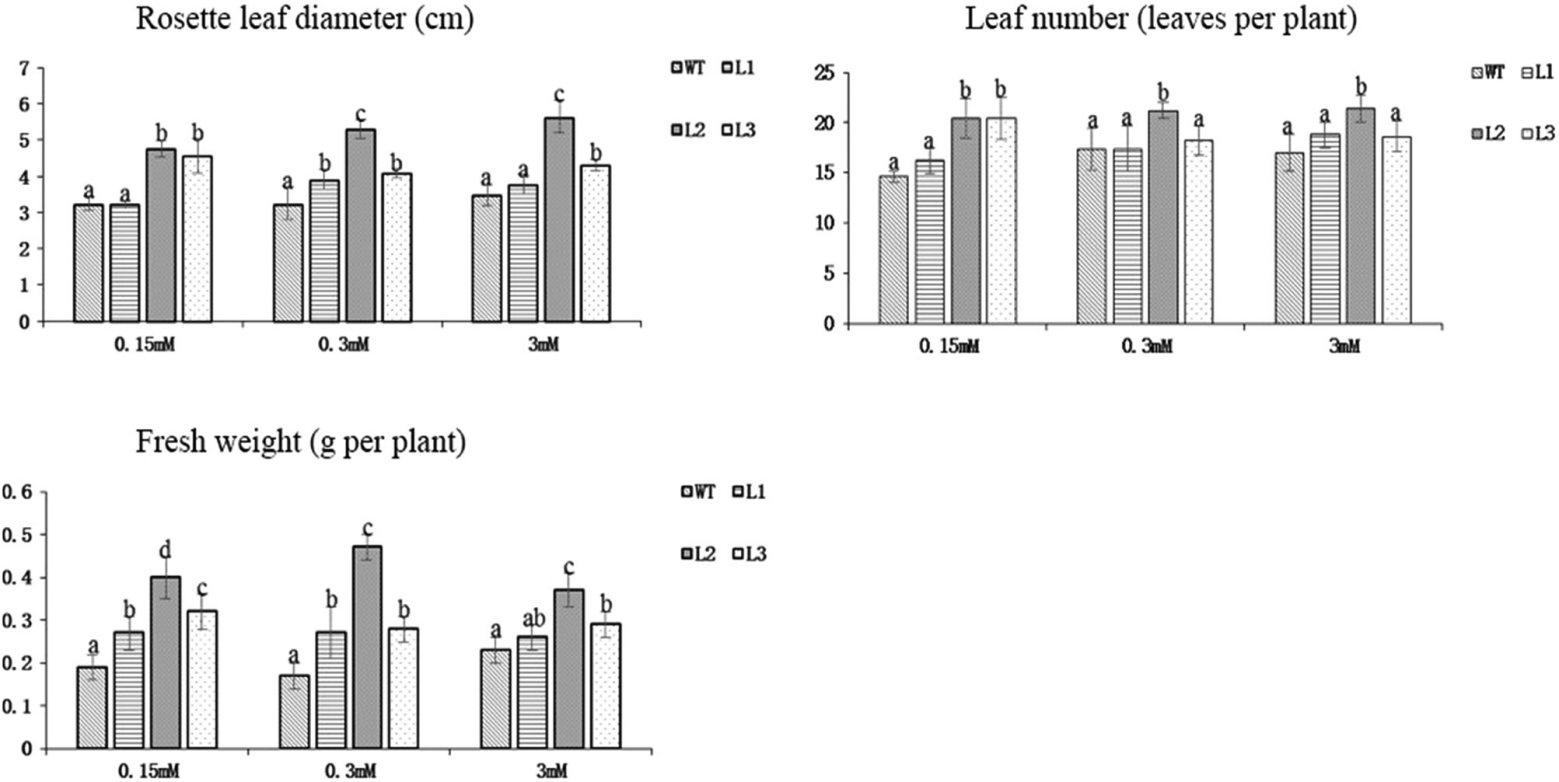
Rosette leaf diameter, leaf number, and fresh weight of *Arabidopsis* transgenic lines and WT plants subjected to 3 different N concentrations for 45 days (*p* < 0.05).

The diameter of the leaves, the number of leaves, and fresh weight were further determined (Figure 17). Compared with that of the WT plants, the diameter of the lotus leaves of the L1 plants increased significantly at a 0.3 mM N concentration, and the diameter of the lotus leaves of the L2 and L3 plants increased significantly at all three N concentrations, which was consistent with the observed phenotypes. The number of leaves in the L2 and L3 plants was significantly different from that of WT plants at the 0.15 mM N concentration, and the L2 plants had significantly more leaves than the WT plants at the 0.3 and 3 mM N concentrations. Although the number of leaves was not significantly different between the L1 and L3 plants and the WT plants, we observed that the fresh weight of the three transformed lines was significantly higher than that of the WT plants at the three concentrations, and the difference between L2 and the WT was the most significant. Taken together, these results indicated that the *PnDof30* gene could improve the growth index of *Arabidopsis thaliana,* especially under low-N levels.

**Figure 18:**
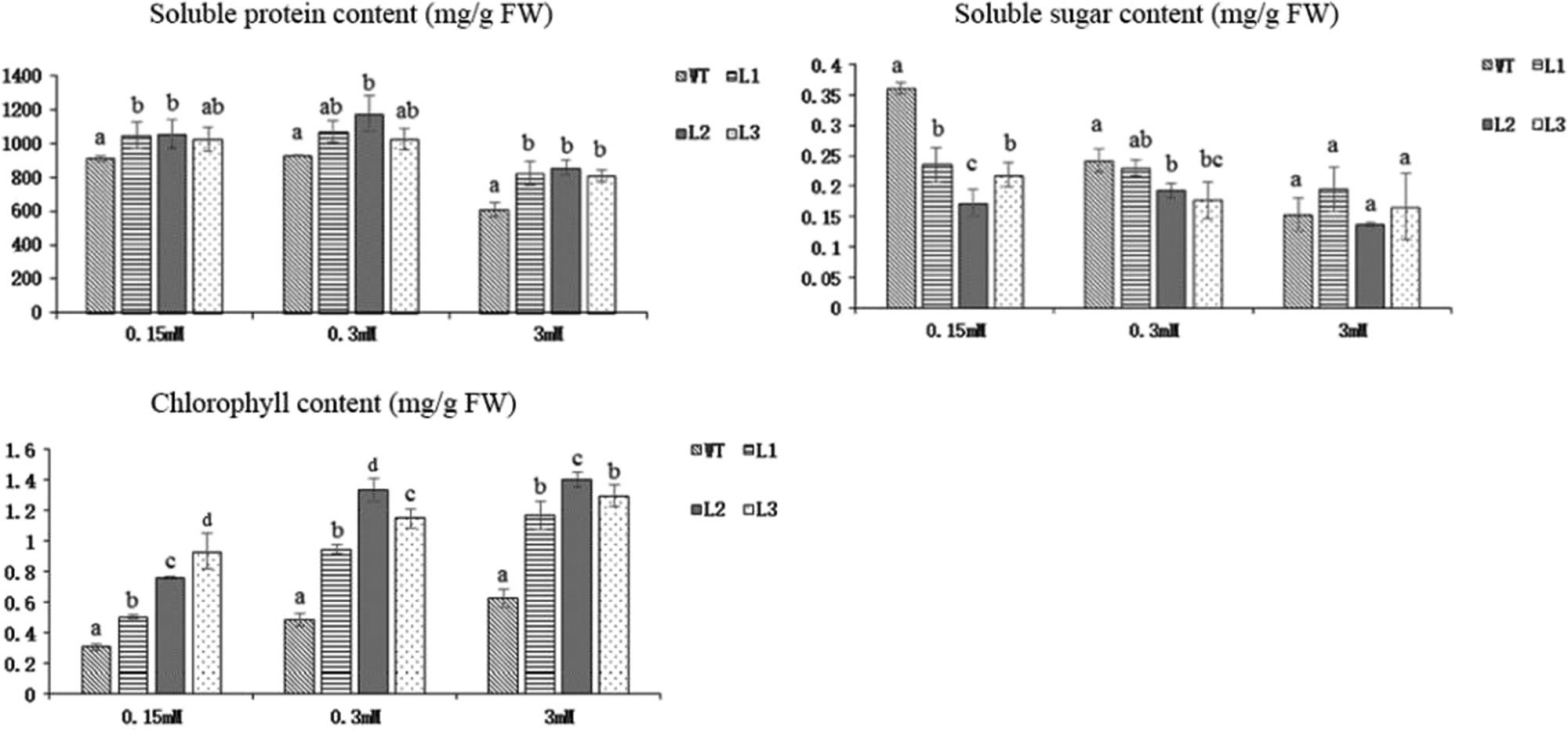
Contents of soluble sugars, soluble proteins, and chlorophyll in *Arabidopsis* transgenic lines and WT plants subjected to 3 different N concentrations for 45 days (*p* < 0.05).

To further evaluate the effect of overexpression of the *PnDof30* gene on the growth of *Arabidopsis thaliana*, we measured the contents of soluble sugars, soluble protein, and chlorophyll (Figure 18). The results showed that the soluble protein contents in the three transgenic lines were higher than those in the WT at the 0.15, 0.3, and 3 mM N concentrations, while the contents in the L3 plants at the 0.15 mM N concentration and in the L1 and L3 plants at 0.3 mM N concentrations were not significantly different from those in the WT, but they were slightly higher. Under the 0.15 mM N concentration, the soluble sugar content in the three transgenic lines decreased significantly; under the 0.3 mM N concentration, the content in the L1 plants decreased slightly, whereas in the L2 and L3 plants, the contents decreased significantly. However, there was no significant difference between the transgenic and WT plants at the 3 mM N concentration. Under the three N concentrations, the chlorophyll content in the transgenic lines was significantly higher than that in the WT plants. The increase in soluble protein content and the decrease in soluble sugar content indicated that the efficiency of N utilization improved and that C skeletons were consumed in the transgenic *Arabidopsis thaliana* lines, which resulted in a decrease in the soluble sugar content and an increase in both the soluble protein content and the chlorophyll content, which effectively promoted photosynthesis and C/N metabolism. Taken together, these results indicate that the *PnDof30* gene can increase the C/N metabolic level in transgenic *Arabidopsis thaliana*, especially under low N levels.

**Figure 19:**
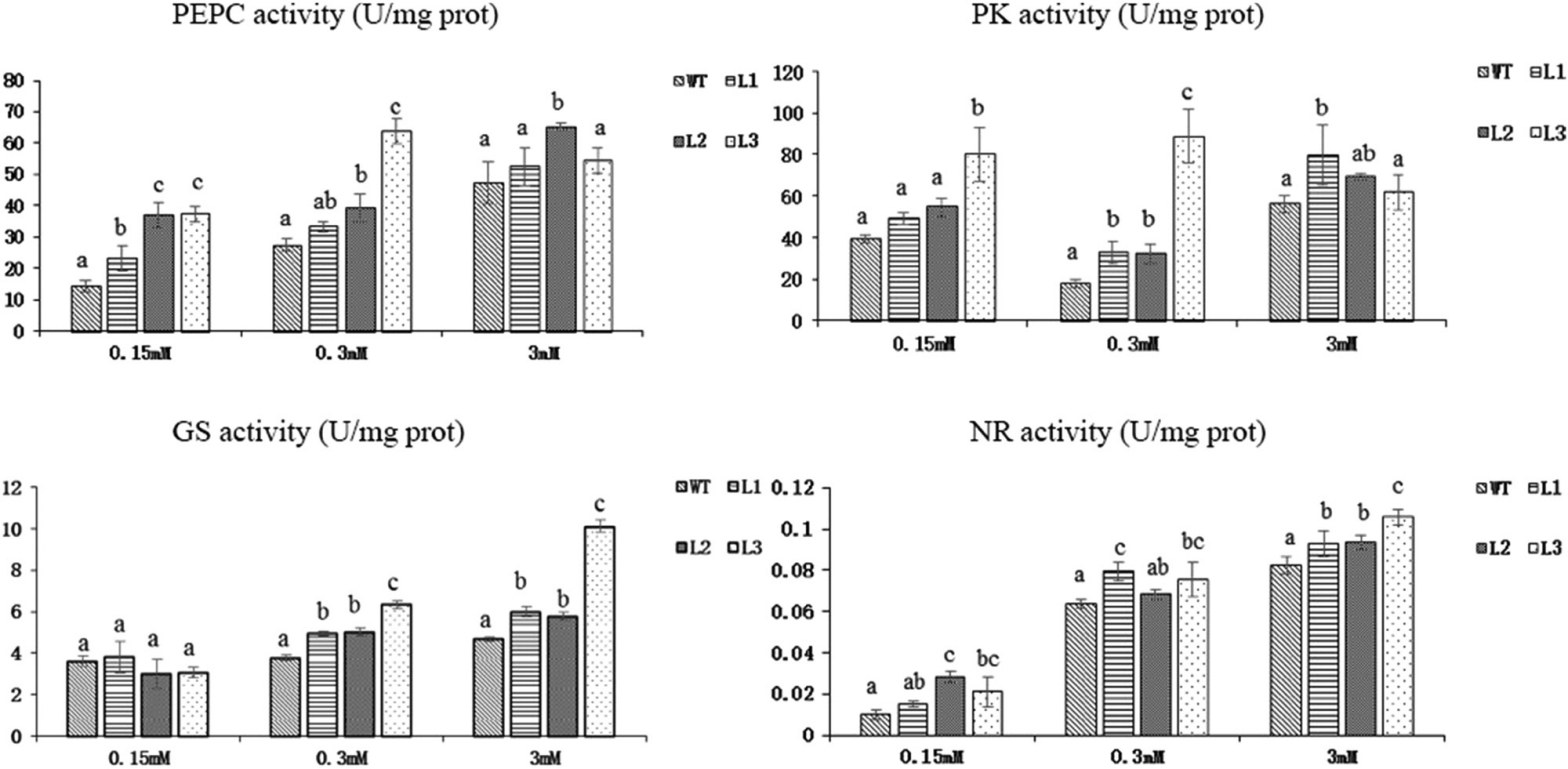
Enzyme activity of PEPC, PK, GS, and NR of *Arabidopsis* transgenic lines and WT under 3 concentrations of N treatment for 45 days (*p* < 0.05).

The activities of the PEPC, PK, GS, and NR enzymes were subsequently determined (Figure 19). According to the PEPC enzyme activity results, the activities in the three transformed lines were significantly higher than that in the WT plants at 0.15 mM N. Under 0.3 mM N, the activity in the L1 plants was slightly higher than that in the WT plants, and that in the L2 and L3 plants was significantly higher than that in the WT plants. Under 3 mM N, the enzyme activity in the L2 plants was significantly higher than that in the WT plants, while the enzyme activity in the L1 and L3 plants did not significantly differ from that in the WT plants.

With respect to PK enzyme activity, the results showed that at 0.15 mM N concentration, the activities in the L1 and L2 plants were slightly higher and that in the L3 plants was significantly higher than that in the WT plants. Under 0.3 mM N, compared with the WT plants, the three transformed lines presented significantly higher PK activity. Under 3 mM N, the activity in the L1 plants was significantly higher than that in the WT plants, and the activities in the L2 and L3 were not significantly different from that in the WT plants.

In terms of GS enzyme activity, the results showed that the three transformed lines did not significantly differ from the WT plants at the 0.15 mM N concentration but that the activity in the former was significantly higher than that in the WT plants at the 0.3 and 3 mM N concentrations.

In terms of NR enzyme activity, the results showed that the activities of the three transgenic lines were significantly higher than those in the WT plants at all three N concentrations.

PEPC and PK are important enzymes involved in the process of C metabolism. The activities of the PEPC and PK enzymes in the transgenic lines increased under low-N conditions, indicating that the metabolism of C increased. Reactions involving GS constitute the first step of ammonium assimilation, and reactions involving NR constitute the first step of nitrate assimilation. The enzyme activities in all transgenic lines improved under low-N conditions. The results of the enzyme activity assay showed that overexpression of *PnDof30* in *Arabidopsis thaliana* could promote C/N assimilation efficiency under low-N conditions.

**Figure 20:**
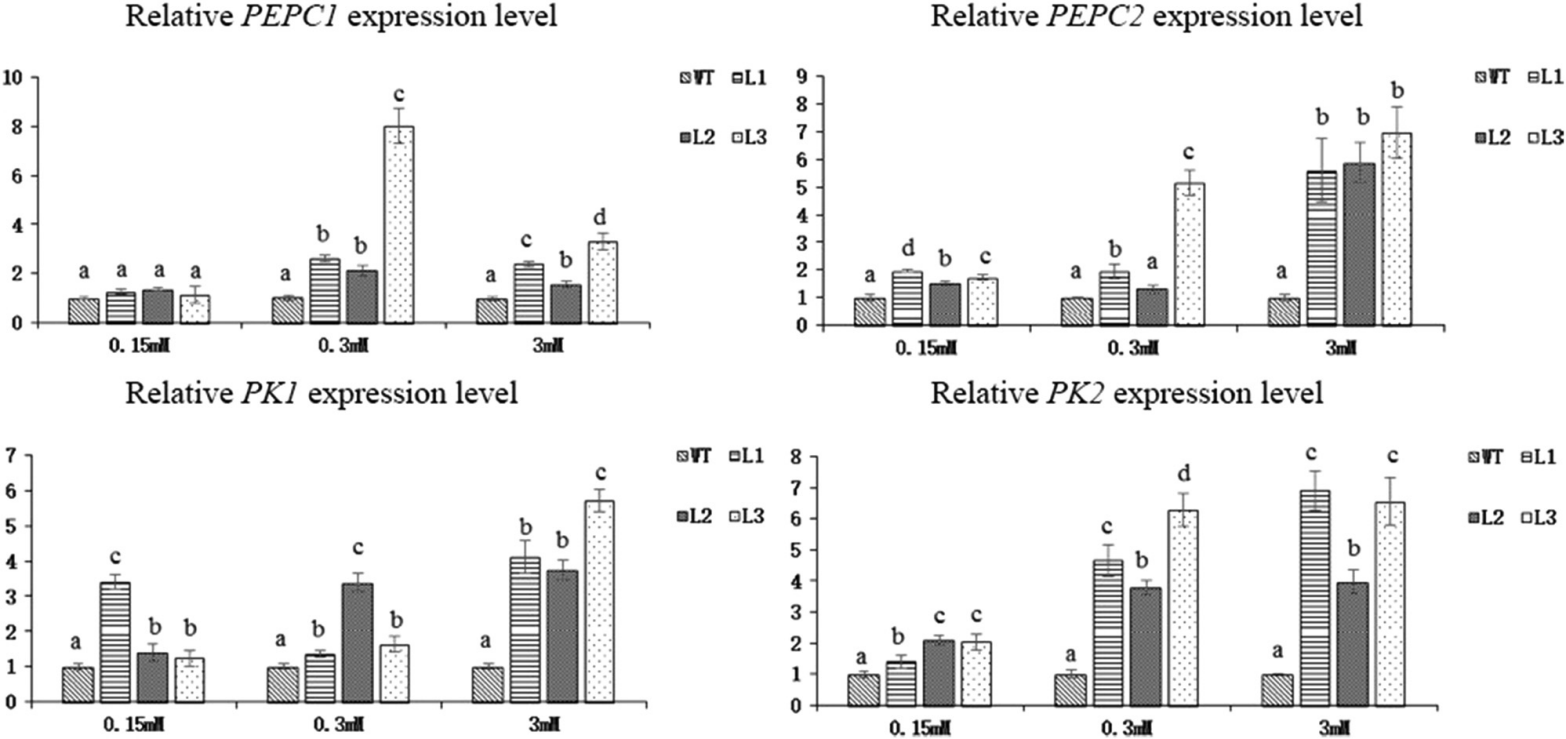
Relative expression levels of the *PEPC* and *PK* genes in *Arabidopsis* transgenic lines and WT plants subjected to 3 different concentrations of N for 45 days (*p* < 0.05).

We selected 13 genes that play a major role in the C/N pathways and measured their relative expression (Figure 20). Under 0.15 mM N, the expression of the *PEPC1* gene in the three transgenic lines was not significantly different from that in the WT plants, but the expression of *PEPC2* significantly increased. Under 0.3 and 3 mM N, the expressions of *PEPC1* and *PEPC2* in the transgenic lines increased. The expressions of *PK1* and *PK2* in the three transgenic lines significantly increased under all three N concentrations. The expression changes of the *PEPC* and *PK* genes were consistent with the results of the PEPC and PK enzyme activities.

**Figure 21:**
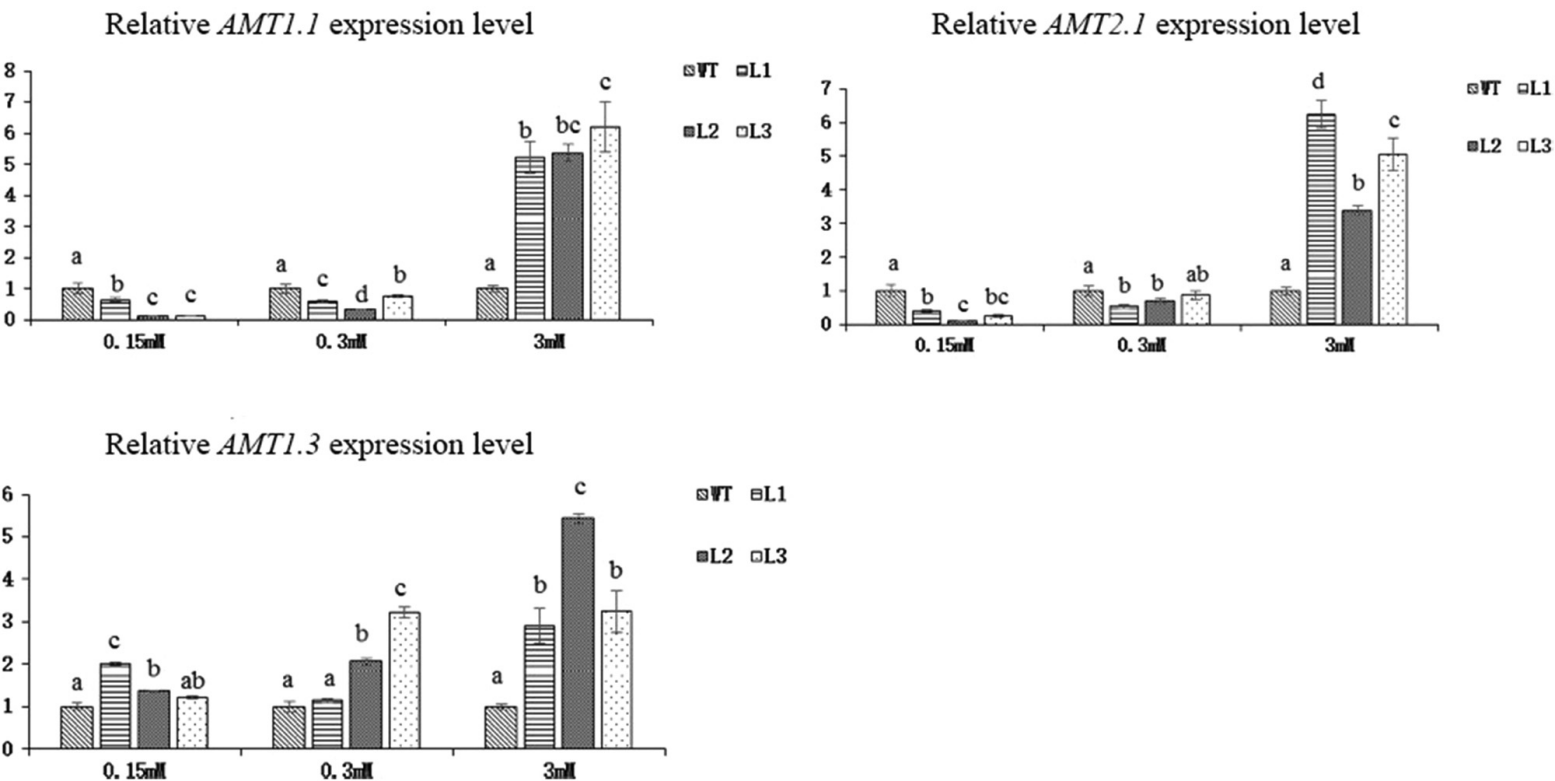
Relative expression levels of *AMT* genes in *Arabidopsis* transgenic lines and WT plants subjected to 3 different concentrations of N for 45 days (*p* < 0.05).

The ammonium transporter (*AMT*) protein data showed that the expression of 3 genes in the 3 transgenic lines significantly increased under 3 mM N (Figure 21). Under low-N conditions, the expressions of *AMT1.1* and *AMT1.2* were downregulated, and that of *AMT1.3* was upregulated. All three genes were major functional genes involved in the high-affinity ammonium transport system in *Arabidopsis thaliana*; however, the contribution of each gene was unknown, so it was uncertain whether ammonium uptake increased in general.

**Figure 22:**
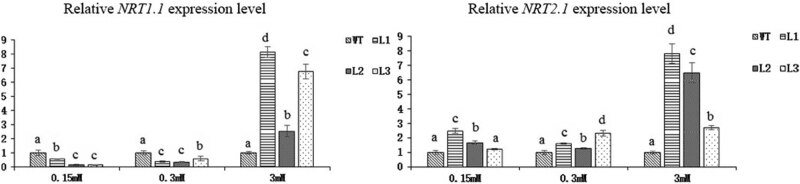
Relative expression levels of *NRT* genes in *Arabidopsis* transgenic lines and WT plants subjected to 3 different concentrations of N for 45 days (*p* < 0.05).

The nitrate transporter (*NRT*) data showed that the expression of two *NRT* genes increased in all three transgenic lines under 3 mM N, but the expression of *NRT1.1* was downregulated and that of *NRT2.1* was upregulated under low-N conditions (Figure 22). Like with the *AMT* gene, it was unclear whether the nitrate transport level improved.

**Figure 23:**
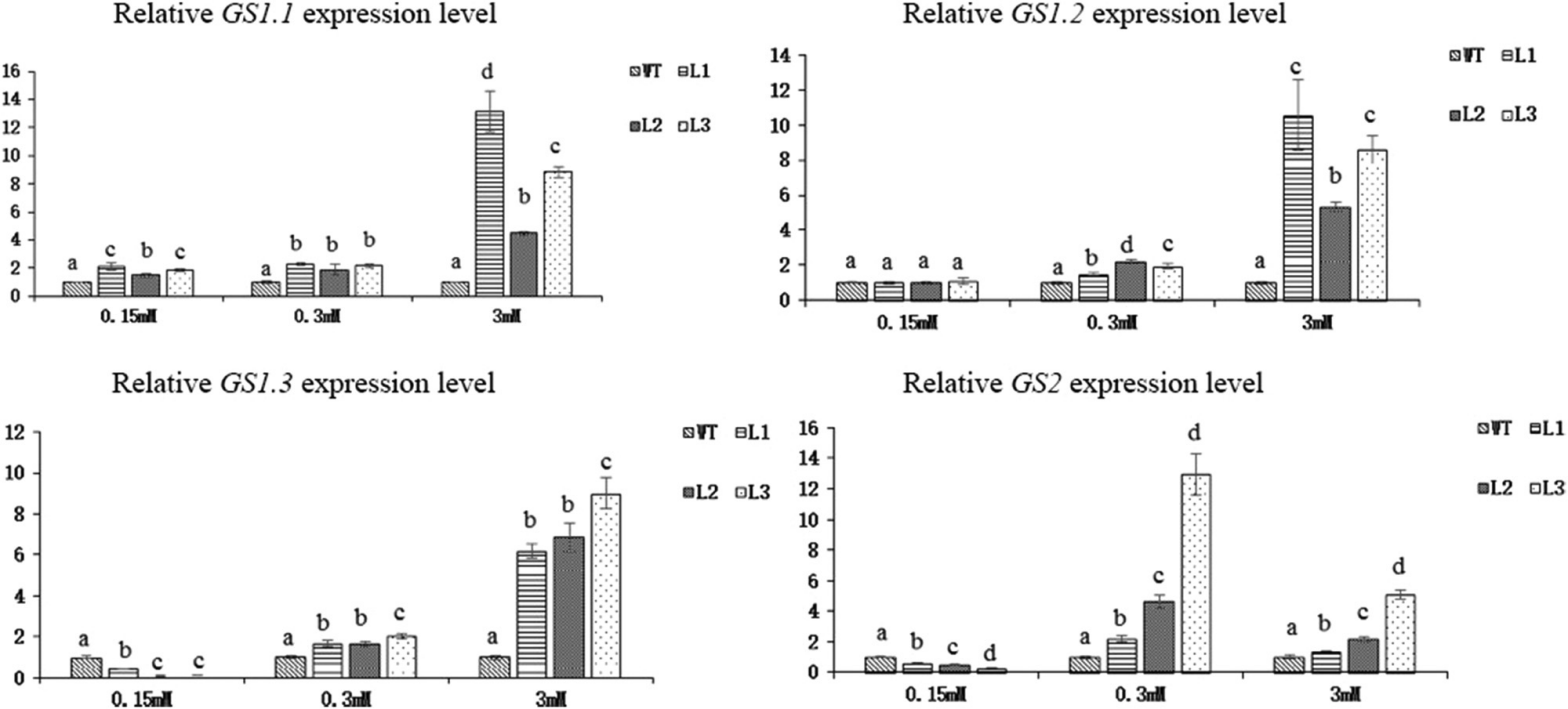
Relative expression levels of *GS* genes in *Arabidopsis* transgenic lines and WT plants subjected to 3 different concentrations of N for 45 days (*p* < 0.05).

The *GS* data showed that the expression of *GS1.1* in the three transgenic lines was significantly higher than that in the WT plants at all three N concentrations (Figure 23). The expressions of *GS1.2*, *GS1.3,* and *GS2* were upregulated under 0.3 and 3 mM N; however, the expression of *GS1.2* was upregulated while the expressions of both GS1.3 and GS2 were downregulated at 0.15 mM N. Under 0.15 mM N, *GS1.2* expression in the transgenic lines was not significantly different from that in the WT plants, and *GS1.3* and *GS2* expression was downregulated. We speculate that 0.15 mM N is the key concentration responsible for *GS1.2*, *GS1.3,* and *GS2* gene expressions. The change in GS gene expression under low-N levels is consistent with the change in GS enzyme activity.

## 4 Discussion

Dof transcription factor family members are specific to plants. Yanagisawa and Izui first identified the gene whose encoded protein contains the Dof domain in 1993 [56]. Dof transcription factors have a variety of functions. Studies have shown that Dof transcription factors regulate the expression of many genes involved in C/N metabolic pathways, promote plant growth, and improve N use efficiency under low-N conditions. However, most of the recent studies on the ability of Dof transcription factors to improve plant N use efficiency have focused on model organisms or crop species with simple genomic backgrounds, such as maize, rice, and *Arabidopsis thaliana*, while the function of Dof transcription factors in forest tree species has rarely been investigated.

In 2006, Yang and Tuskan identified 41 *Dof* genes from *Populus trichocarpa* based on the V1.0 database [57] (Appendix Table A1). With the release of the *Populus trichocarpa* V2.2 database and the development of bioinformatics technology, Wang re-identified the Dof family members in *Populus trichocarpa* in 2017. Although the number of members identified was still 41, the content and depth of the study were improved, but the focus was mainly on osmotic stress [58]. Based on the new *Populus trichocarpa* V3.0 database, 44 members of the *Dof* gene family were identified in this study, three more than the total number previously identified. Except for the PtDof29, all the members contain a highly conserved Dof domain that includes four cysteines that constitute a zinc-finger structure, which is an important component of transcription factors. We divided all the members into four subfamilies according to the evolutionary relationships between the genes. We identified nine pairs of genes originating from homologous recombination events by performing a phylogenetic analysis and using chromosome mapping data. Gene expression pattern analysis revealed that most of the Dof genes were expressed in young leaves, stems, and roots.

In this study, the Dof genes of *Populus trichocarpa* after N treatment were screened via qRT-PCR. Finally, we screened three genes, *PnDof19*, *PnDof20*, and *PnDof30*, which responded to changes in N and whose expression changed with changes in N concentration.

We cloned the CDSs of the *PnDof19*, *PnDof20*, and *PnDof30* genes from *Populus trichocarpa* cDNA and fused the sequences to the GFP gene for subcellular localization experiments. The results showed that all three transcription factors were localized in the nucleus, which was consistent with the localization of Dof proteins in other species [54,55].

N treatment was carried out on homozygous lines of *PnDof30* transgenic *Arabidopsis thaliana*. The results showed that the transgenic *Arabidopsis thaliana* plants grew better than the WT plants under low-N conditions; the soluble protein and chlorophyll contents significantly increased, while the soluble sugar content significantly decreased. These results were consistent with those of Yanagisawa et al.’s research [31]. PEPC catalyzes the reaction of phosphoenolpyruvic acid with HCO_3_^−^ to form oxaloacetic acid, a supplemental substrate of the tricarboxylic acid cycle [59]. PK catalyzes the production of pyruvic acid from phosphoenolpyruvate. These two enzymes are key enzymes involved in the process of C assimilation. Both the gene expression and enzyme activity of PEPC and PK significantly increased in the transgenic lines, indicating that overexpression of *Dof* genes increased the level of C metabolism. The reduction of nitrate to nitrite catalyzed by NR is the first step in nitrate assimilation, and NR gene expression and enzyme activity significantly increased. AMT and NRT are important transporters of inorganic N absorbed by plant roots. The expression of some of the major genes encoding both AMT and NRT was upregulated, and the expression of some was downregulated. Although it was unclear which gene contributes more to the uptake of inorganic N, a higher soluble protein content meant that transgenic *Arabidopsis* might have a higher overall N uptake efficiency. GS1.3 and GS2 expressions and enzyme activities were downregulated under 0.15 mM N, but the opposite results were observed under 0.3 mM N. We speculated that the 0.15 mM N concentration was the key regulatory concentration for GS1.3 and GS2.

The innovation of this study is that, first, we re-identified the Dof family of *Populus trichocarpa*, which has three more members than in previous studies; second, we cloned three Dof members in *Populus simonii: PnDof19, PnDof20, and PnDof30*; and third, in 2013, Lin transformed maize ZmDof1 into poplar and found that plant growth indicators and N assimilation were not improved at low-N levels. It is speculated that maize ZmDof1 is not suitable for poplar, a forest plant. The gene *PnDof30*, which can improve the growth index and C/N metabolism-related physiological index of *Arabidopsis* under low-N levels, was cloned by itself. This gene can be used as an important alternative tool to improve the growth state of poplar under a low-N environment.

## 5 Conclusion

This study focused on the expression of Dof gene in roots and leaves by N treatment of *Populus nigra*, and whether they were induced by N. According to the Dof gene expression heat map constructed from the EFP database in the bioinformatics chapter, we found 13 genes with high expression levels in leaves and roots: PtDof4/10/12/14/19/20/27/28/32/34/36/38/43; On the heat map made by the FPKM database, there are six genes with high expression in roots and leaves at the same time PtDof5/10/19/20/30/32; up to 16 genes were induced by N in leaves in experiments with supply interruption and restoration: PtDof6/8/10/11/20/21/23/24/26/27/28/30/32/35/37/40, while there are only two genes induced by N in roots: PtDof3/16, which indicates that the expression of Dof gene that plays a role in leaves is mostly induced by nitrogen, while the expression of Dof gene in roots is composed of type expression.

Based on the above expression data, we selected six genes, PtDof10/19/30 (subfamily I), PtDof32 (subfamily II), PtDof12 (subfamily III), and PtDof20 (subfamily IV), as candidate genes for functional studies. Through repeated gene cloning experiments, we finally cloned the three genes *PnDof19*, *PnDof20*, and *PnDof30* for subsequent experiments. The Dof gene of *P. nigra* that responds to N was screened out by quantitative PCR. These three genes responded to changes in N, and their expression varied with N concentrations.

Through the method of molecular biology, we cloned *PnDof19*, *PnDof20*, and *PnDof30* genes. The length of the open reading frame of the *PnDof19* gene is 744 bp, encoding 248 AAs; the length of the ORF region of the *PnDof20* gene is 969 bp, encoding 323 AAs; The ORF region of the *PnDof30* gene is 1,071 bp in length and encodes 357 AAs. After cloning, we first fused these genes with the green fluorescent protein gene GFP and expressed them in the onion lower epidermis by gene gun transient transformation to explore the subcellular localization of these three transcription factors localized in the nucleus.

To further explore whether these three Dofs can promote N assimilation and regulate C/N metabolism, considering that *Populus simonii* × *Populus nigra* belongs to the Aigeiros segment, the transformation is difficult, so we constructed these three genes into the plant overexpression vector pROK2, and tried to transform *Arabidopsis thaliana*. After many attempts, only *PnDof30* was successfully transformed into *Arabidopsis*. After repeated verification and screening, a homozygous line for follow-up research was successfully obtained.

According to the above reviews, the overexpression of *PnDof30*, a member of the Dof family in *Populus simonii* × *Populus nigra*, could promote the growth of *Arabidopsis* and increase the level of C/N metabolism under low-N conditions. Therefore, the *Dof* gene in *Populus simonii* × *Populus nigra* may be used as an important candidate to improve the growth of poplar under low-N conditions.

## 6 Author summary

In this study, we treated *Populus simonii × Populus nigra* with N, and focused on observing the expression of Dof gene in roots and leaves, and testing whether they are induced by N. Next we cloned the three genes that respond to N and constructed them into a plant expression vector. Finally, one of the genes was successfully expressed. In the subsequent low-N treatment, it was also confirmed that this gene can promote plant growth and increase the level of C/N metabolism.

Compared with the predecessors, the results of this study show that the genes of the Dof family of *Populus trichocarpa* have increased significantly. In addition, we also cloned three of the genes with good results. In addition, we cloned the gene *PnDof30* from the small black poplar itself that can improve the growth indicators of *Arabidopsis thaliana* and the physiological indicators related to C/N metabolism under low-N levels. This gene can be used as a way to improve the growth status of poplars in low-N environments.

## Appendix

**Table A1:** Dof family members of *Populus trichocarpa* in this study and their members identified in 2017

Name	Phytozome	Member in 2017	Phytozome
*PtDof01*	Potri.001G086400	*PtrDof36*	POPTR_0001s11130
*PtDof02*	Potri.001G238400	*PtrDof32*	POPTR_0001s24540
*PtDof03*	Potri.002G070700	*PtrDof16*	POPTR_0002s07150
*PtDof04*	Potri.002G129600	*PtrDof17*	POPTR_0002s13100
*PtDof05*	Potri.002G174300	*PtrDof33*	POPTR_0002s17490
*PtDof06*	Potri.003G034200	*PtrDof30*	POPTR_0003s02890
*PtDof07*	Potri.003G144500	*PtrDof31*	POPTR_0003s14450
*PtDof08*	Potri.004G038800	*PtrDof27*	POPTR_0004s03900
*PtDof09*	Potri.004G046100	*PtrDof2*	POPTR_0004s04590
*PtDof10*	Potri.004G046600		
*PtDof11*	Potri.004G056900	*PtrDof1*	POPTR_0004s05580
*PtDof12*	Potri.004G121800	*PtrDof22*	POPTR_0004s12120
*PtDof13*	Potri.005G131600	*PtrDof34*	POPTR_0005s13990
*PtDof14*	Potri.005G134200	*PtrDof4*	POPTR_0005s14080
*PtDof15*	Potri.005G149100	*PtrDof5*	POPTR_0005s19310
*PtDof16*	Potri.005G188900		
*PtDof17*	Potri.006G084200	*PtrDof35*	POPTR_0006s08440
*PtDof18*	Potri.006G202500	*PtrDof18*	POPTR_0006s21700
*PtDof19*	Potri.007G036400	*PtrDof23*	POPTR_0007s11790
*PtDof20*	Potri.007G038100	*PtrDof6*	POPTR_0007s11620
*PtDof21*	Potri.007G058200	*PtrDof26*	POPTR_0007s09520
*PtDof22*	Potri.008G055100	*PtrDof39*	POPTR_0008s05520
*PtDof23*	Potri.008G087800	*PtrDof37*	POPTR_0008s08740
*PtDof24*	Potri.009G029500	*PtrDof40*	POPTR_0009s03490
*PtDof25*	Potri.010G167600	*PtrDof38*	POPTR_0010s17480
*PtDof26*	Potri.010G205400	*PtrDof14*	POPTR_0010s21240
*PtDof27*	Potri.011G047500	*PtrDof28*	POPTR_0011s04730
*PtDof28*	Potri.011G054300	*PtrDof12*	POPTR_0011s05410
*PtDof29*	Potri.011G054400		
*PtDof30*	Potri.011G055600	*PtrDof13*	POPTR_0011s05450
*PtDof31*	Potri.011G065900	*PtrDof7*	POPTR_0011s07400
*PtDof32*	Potri.012G018700	*PtrDof20*	POPTR_0012s02570
*PtDof33*	Potri.012G063800	*PtrDof19*	POPTR_0012s12670
*PtDof34*	Potri.012G081300	*PtrDof21*	POPTR_0012s08280
*PtDof35*	Potri.013G066700	*PtrDof24*	POPTR_0013s06290
*PtDof36*	Potri.014G036600	*PtrDof15*	POPTR_0014s03590
*PtDof37*	Potri.014G100900	*PtrDof29*	POPTR_0014s09640
*PtDof38*	Potri.015G009300	*PtrDof41*	POPTR_0015s01160
*PtDof39*	Potri.015G048300	*PtrDof9*	POPTR_0015s03520
*PtDof40*	Potri.015G077100	*PtrDof10*	POPTR_0015s08810
*PtDof41*	Potri.016G069300	*PtrDof11*	POPTR_0016s07000
*PtDof42*	Potri.017G084600	*PtrDof25*	POPTR_0017s12080
*PtDof43*	Potri.019G040700	*PtrDof8*	POPTR_0019s05720
*PtDof44*	Potri.T146000	*PtrDof3*	POPTR_0005s21130
